# Albuminuria, but not eGFR, tracks diabetic retinopathy severity and retinal ischemia: population-based discovery, clinical replication, and OCTA evidence

**DOI:** 10.3389/fendo.2026.1843774

**Published:** 2026-06-19

**Authors:** Xiaojing Zha, Yizheng Zhang, Yuqi Ren, Zhiyong Meng, Chenming Zhang

**Affiliations:** 1 Department of Ophthalmology, Shaowu Municipal Hospital, Shaowu, Fujian, China; 2 Department of Ophthalmology, Shaowu City Traditional Chinese Medicine Hospital, Shaowu, Fujian, China; 3 Department of Oncology, Shengli Clinical Medical College of Fujian Medical University, Fujian Provincial Hospital, Fuzhou University Affiliated Provincial Hospital, Fuzhou, China

**Keywords:** albuminuria, diabetic retinopathy, eGFR, NHANES, OCTA, urine albumin-to-creatinine ratio

## Abstract

**Background:**

We investigated whether albuminuria is more consistently associated with diabetic retinopathy (DR) severity than estimated glomerular filtration rate (eGFR) and whether it corresponds to an ischemic retinal phenotype on optical coherence tomography angiography (OCTA).

**Methods:**

This two-part cross-sectional study first analyzed 949 adults with diabetes from the National Health and Nutrition Examination Survey (NHANES) to evaluate the associations of albumin-to-creatinine ratio (ACR) and eGFR with the DR outcomes. An independent single-center cohort of 168 adults with type 2 diabetes, including a nested 134-participant OCTA subset, was then used to examine the associations of urine ACR (UACR) and eGFR with the clinical DR endpoints and retinal perfusion metrics.

**Results:**

In the NHANES, each doubling of ACR was independently associated with higher odds of AnyDR and SevereDR, with stronger association for SevereDR (odds ratio, OR = 1.30), whereas eGFR showed no independent association. In the clinical cohort, each doubling of UACR was associated with higher odds of moderate-or-worse DR and vision-threatening diabetic retinopathy (VTDR) (OR = 2.86 and 2.70, respectively), while eGFR remained non-significant. In the OCTA subset, a higher UACR was associated with greater widefield non-perfusion and lower macular perfusion density, whereas eGFR was not associated with either OCTA endpoint.

**Conclusions:**

Across the studied cohorts, albuminuria was consistently associated with more severe DR and an ischemic OCTA phenotype, whereas eGFR did not show an independent association within the observed kidney function range. Spot urine ACR testing may serve as a practical biomarker for stratifying retinal microvascular ischemia risk.

## Introduction

Diabetic retinopathy (DR) remains a leading cause of vision loss among adults with diabetes. Because retinal injury may mirror broader systemic microvascular damage, accessible systemic markers that track DR burden could improve risk stratification, refine ophthalmic surveillance, and inform a more integrated view of end-organ injury in diabetes (1, 2).

The kidney is a particularly relevant comparator organ. DR and diabetic kidney disease commonly coexist, and the retina and glomerulus share susceptibility to endothelial injury, basement membrane remodeling, and capillary dysfunction. However, the two renal measures most widely used in practice, i.e., albuminuria and estimated glomerular filtration rate (eGFR), capture different aspects of the disease biology. Albuminuria is more closely linked to capillary barrier failure and endothelial leak, whereas eGFR is a broader functional index influenced by nephron reserve, hemodynamics, age, and muscle mass and may remain preserved despite ongoing retinal microvascular injury (3–6).

These distinctions may help explain why prior studies have reported inconsistent associations between renal measures and DR severity: albuminuria and eGFR were not always evaluated in the same adjusted model, and the retinal outcomes were often defined only by conventional fundus-based grading rather than direct measures of capillary perfusion. This leaves uncertain whether the renal signal associated with more severe DR also corresponds to a quantitatively ischemic retinal phenotype. Optical coherence tomography angiography (OCTA) offers an opportunity to address this gap by directly quantifying retinal capillary non-perfusion and perfusion loss *in vivo* (7–10).

Accordingly, we designed a staged two-part study across complementary settings. We first used the National Health and Nutrition Examination Survey (NHANES) 2005–2008 as a nationally representative discovery cohort to examine whether albuminuria is independently associated with DR severity after accounting for eGFR and major clinical covariates. We then examined an independent single-center ophthalmic specialty clinic cohort to assess replication in a higher-acuity clinical setting and to determine whether the same renal signal extends to OCTA-defined retinal ischemia. We hypothesize that higher albuminuria, measured as albumin-to-creatinine ratio (ACR) in the NHANES and urine ACR (UACR) in part 2, but not eGFR, would be independently associated with more severe DR and with a more ischemic OCTA phenotype.

## Methods

### Study overview

This integrated investigation used a staged two-part cross-sectional design. Part 1 is a nationally representative NHANES discovery analysis that examined whether albuminuria and eGFR are independently associated with DR in the US diabetes population. Part 2 evaluated the same renal–retinal relationship in an independent consecutive single-center ophthalmic specialty clinic cohort and, within a nested OCTA bridge set, assessed whether albuminuria maps to a quantitatively ischemic retinal phenotype. The two parts were analyzed separately because they differed in sampling frame, outcome definitions, imaging platforms, and estimands. Accordingly, the interpretation emphasized directional and biologic concordance rather than pooled estimation. Throughout the manuscript, we use albuminuria as the generic term for the shared renal signal and retain cohort-specific labels, i.e., ACR in the NHANES and UACR in part 2, within cohort-specific analyses.

### Part 1: NHANES discovery cohort

#### Study design, data source, and ethical considerations

Part 1 used publicly available data from the NHANES 2005–2006 and 2007–2008. In keeping with standard NHANES analytic practice, the demographic, diabetes questionnaire, examination, laboratory, and ophthalmology files were linked by the respondent sequence number (SEQN). These two adjacent cycles were selected as both included the retinal photography component required for DR grading. NHANES uses a stratified, multistage probability design to represent the civilian, non-institutionalized US population, with standardized household interviews, mobile examination center (MEC) examinations, laboratory testing, and ophthalmic imaging performed under centrally harmonized protocols. All participants provided written informed consent, and each cycle was approved by the National Center for Health Statistics Research Ethics Review Board (11).

#### Study population and diabetes definition

**Figure 1:**
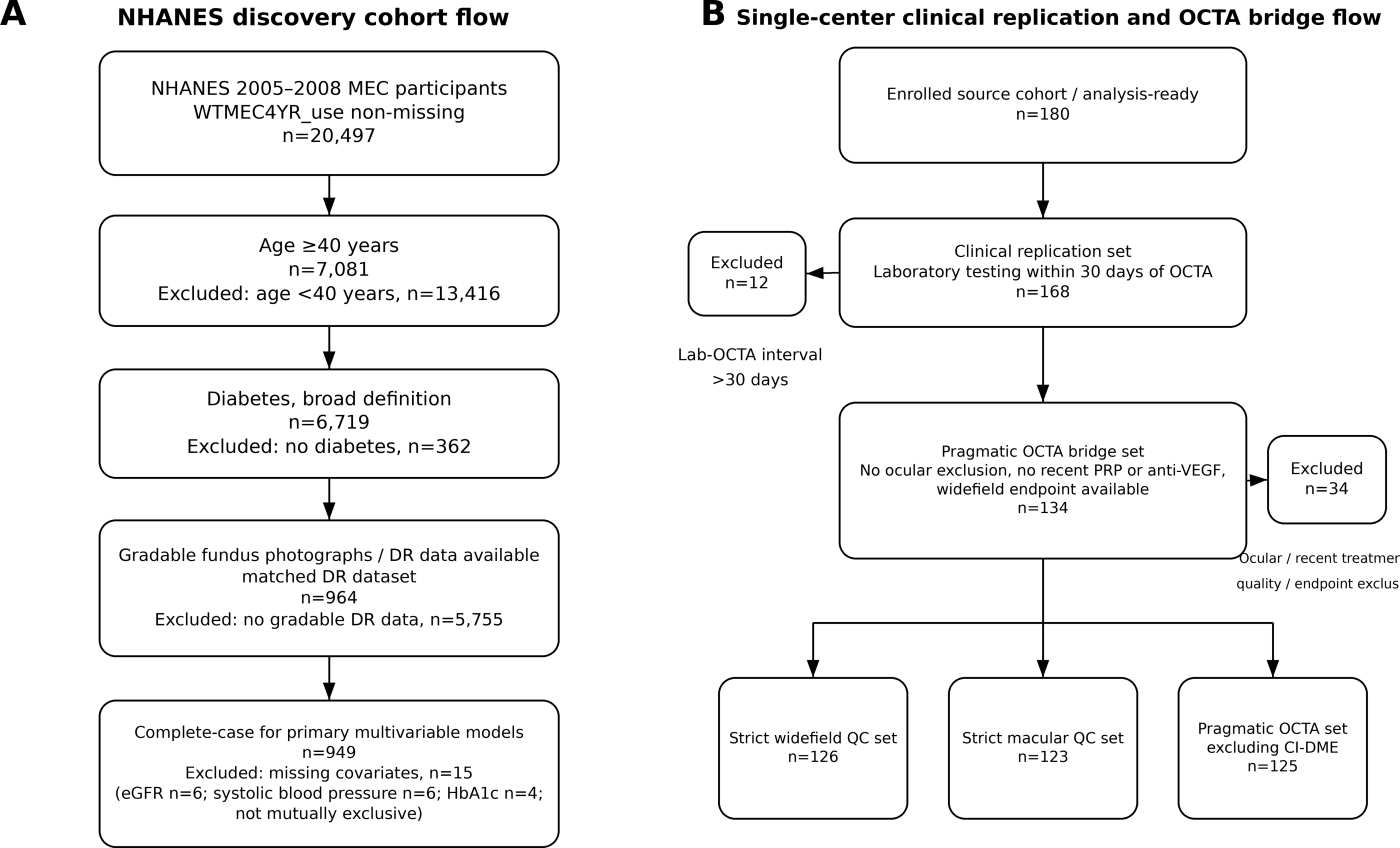
Study flow diagrams. **(A)** shows the NHANES discovery cohort flow. NHANES 2005-2008 MEC participants with nonmissing four-year MEC weights entered a stepwise discovery workflow requiring age ≥40 years, the broad diabetes definition, and gradable fundus photographs linked to DR grading. This yielded an analytic NHANES cohort of 964 participants for descriptive and outcome analyses; after minimal and overlapping complete-case exclusions for eGFR, systolic blood pressure, and HbA1c, 949 participants contributed to the fully adjusted survey-weighted regression models. **(B)** shows the single-center clinical replication and OCTA bridge flow, including progression from the 180-participant enrolled source cohort to the 168-participant clinical replication set after exclusion of 12 participants whose closest laboratory panel was more than 30 days from OCTA, and then to the 134-participant pragmatic OCTA bridge set after ocular, treatment-related, and imaging exclusions. Because recent PRP or anti-VEGF treatment, vitrectomy, dense media opacity, and noninterpretable scans were excluded, the pragmatic OCTA set is best interpreted as capturing the pre-destructive-intervention retinal microvascular state rather than the full spectrum of treated late-stage disease.

Eligible NHANES participants were MEC examinees aged 40 years or older, as retinal imaging is performed only in this age range, who met the study diabetes definition, had gradable retinal photographs with linked DR grading, and had urine albumin, urine creatinine, serum creatinine, and prespecified covariates available for the primary models. The analytic sample therefore was anchored to the retinal imaging denominator and then restricted only minimally for complete case regression. Diabetes was defined by self-reported physician diagnosis, current insulin use, current oral hypoglycemic medication use, or glycated hemoglobin (HbA1c) 6.5% or greater. We did not require fasting plasma glucose because it is available only in the fasting subsample and would have forced the use of fasting subsample weights, materially shrinking the retinal photography cohort and changing the survey-weighted target population. Questionnaire responses coded as “Refused” or “Don’t know” were treated as missing. **Figure 1A**; **Supplementary Table 4** show the derivation of the NHANES analytic cohort: 964 participants contributed descriptive analyses and 949 contributed the fully adjusted complete case models.

#### Retinal outcome ascertainment

Retinal photographs were obtained in the MEC using two 45-degree non-mydriatic digital images per eye under the NHANES ophthalmology protocol and were graded centrally using the NHANES-modified Early Treatment Diabetic Retinopathy Study framework. Participant-level DR status was based on the worse eye severity grade to mirror the participant-level design used in part 2. We prespecified two binary retinal outcomes spanning overall presence and more advanced disease—AnyDR, defined as worse eye severity grade 2 or higher, and SevereDR, defined as worse eye severity grade 3 or higher—corresponding to moderate or severe non-proliferative DR or proliferative DR. These NHANES outcomes were retained as the discovery-stage endpoints, after which part 2 examined the same renal–retinal signal against clinically anchored ICDR-based endpoints in an independent clinical cohort (12, 13).

#### Kidney exposures and covariates

Kidney exposures were ACR and eGFR. ACR was derived from the urine albumin and urine creatinine measured in a single random spot urine specimen collected in the MEC, in parallel with the spot urine UACR design in part 2. The serum creatinine from the public laboratory files was used to calculate the eGFR with the 2021 Chronic Kidney Disease Epidemiology Collaboration (CKD-EPI) creatinine equation without a race coefficient and is reported in milliliters per minute per 1.73 m (2). In the primary models, albuminuria was modeled on the base-2 logarithmic scale; therefore, the effect estimates represent the odds ratio (OR) per doubling of the ACR. Clinical ACR categories of <30, 30–299, and 300 mg/g or higher were reserved for supportive descriptive and clinical interpretation analyses, and natural-log ACR was retained for exploratory visualization. The prespecified adjustment variables were age, sex, race/ethnicity, body mass index (BMI), systolic blood pressure, and HbA1c. Diabetes duration was not included in the primary NHANES models because the study diabetes definition intentionally combined participants identified from questionnaire data with participants newly identified from HbA1c, leaving no uniformly measured, design-consistent duration variable across the full retinal photography cohort. Requiring self-reported age at diagnosis would have selectively reduced the analytic sample and mixed systematically measured and recalled disease timing. Consequently, cross-cohort comparisons were intended to assess directional and biological concordance rather than direct equivalence of effect sizes because the NHANES and part 2 models could not be fully harmonized for diabetes duration. Low-density lipoprotein cholesterol and triglycerides were also excluded from the primary NHANES models as these are available only in the fasting morning subsample and would require fasting subsample weights, thereby changing both the sample size and the survey-weighted target estimand. Systolic blood pressure was summarized as the mean of the available standardized MEC readings. For estimate stability, race/ethnicity was collapsed into four categories in the primary models and retained in its original five-category NHANES coding in the sensitivity analyses (14).

#### Statistical analysis

All NHANES analyses followed the National Center for Health Statistics analytic guidance for multicycle survey data. Because the merged analysis relied on MEC examination, laboratory, and retinal photography variables rather than fasting subsample analytes, we used a derived 4-year MEC examination weight (WTMEC4YR_use, defined as 0.5 × WTMEC2YR within each cycle for 2005–2006 and 2007–2008) together with SDMVSTRA (stratum) and SDMVPSU (primary sampling unit) to account for unequal selection probabilities, clustering, and stratification. Variance estimation used Taylor series linearization. Weighted estimates are therefore interpretable as nationally representative estimates for US adults with diabetes aged 40 years or older who attended the MEC and had gradable retinal photographs. Continuous variables are reported as survey-weighted mean ± standard errors and categorical variables as survey-weighted percentages, with unweighted denominators shown for transparency (15).

Primary NHANES inference used survey-weighted logistic regression for AnyDR and SevereDR, with design-corrected standard errors and 95% confidence intervals. The primary models jointly included log2-transformed ACR and eGFR plus the prespecified covariates because the *a priori* question was whether albuminuria retained an association with DR independent of the filtration function. Supportive analyses replaced continuous ACR with clinical categories and tested ordered trend, refit adjusted models using the original five-level RIDRETH1 race/ethnicity variable, and used restricted cubic splines with an exploratory piecewise model to visualize functional form. Missingness was minimal: eGFR was missing in six participants, systolic blood pressure in six, and HbA1c in four, with overlap. Complete case analysis was therefore prespecified, and no imputation was performed. Because combined missingness affected fewer than 1% of the analytic cohort, meaningful bias from the complete case analysis was considered unlikely. All statistical analyses were performed using R software, version 4.3.1 (R Foundation for Statistical Computing, Vienna, Austria). Complex survey data analyses were conducted using the *survey* package, and restricted cubic splines were fitted using the *rms* package. All tests were two-sided. Exact *p*-values are reported when *p* ≥ 0.001 and smaller values as *p* < 0.001.

### Part 2: Single-center clinical replication and OCTA bridge

#### Study design

Part 2 used a de-identified single-center ophthalmic specialty clinic cross-sectional clinical dataset assembled for two linked purposes: external replication of the NHANES albuminuria–DR association and mechanistic bridging to the OCTA-derived retinal microvascular phenotypes. Adults with type 2 diabetes were enrolled consecutively from September 2025 through March 2026, and the OCTA index visits occurred from October 2025 through March 2026. The source workbook was structured at participant, eye, and scan levels and linked by a common study identifier, with a query resolution log that allowed each final analytic variable to be traced back to its source record. The enrolled source cohort comprised 180 adults. All 180 subjects already had participant-level DR grading, UACR, serum creatinine and eGFR, HbA1c, and blood pressure recorded; therefore, part 2 derivation was driven by prespecified timing and image eligibility rules rather than missing core covariates. For the clinical replication analysis, the laboratory values were anchored to the OCTA index visit. Same-day results were preferred; otherwise, the closest available panel within 30 days was accepted. A total of 12 otherwise complete participants had the nearest laboratory panel more than 30 days from the OCTA, ranging from 32 to 57 days, and were excluded, leaving 168 participants.

The pragmatic OCTA bridge set was nested within this 168-participant clinical replication set. In addition to meeting the laboratory window criterion, each participant had to contribute one prespecified analysis eye with the fixed OCTA acquisition protocol and the primary widefield endpoint available. Eyes were excluded from the primary OCTA analysis for other retinal vascular disease, major media opacity likely to impair interpretation, including dense cataract or vitreous hemorrhage, high or pathologic myopia, prior vitrectomy, or recent retinal intervention expected to materially alter the OCTA metrics, defined as anti-vascular endothelial growth factor (anti-VEGF) treatment within 3 months or panretinal photocoagulation (PRP) within 6 months. Eyes were also excluded for non-interpretable scans due to poor signal, major motion artifact, marked decentration, or segmentation failure. Center-involving diabetic macular edema (CI-DME) was retained in the main OCTA analysis because it is clinically relevant, but was prespecified for the sensitivity analysis as it can distort layer segmentation and perfusion measurements. Participant flow is summarized in **Figure 1B**; **Supplementary Tables 6**, **7**. The exclusion patterns are also shown in **Supplementary Figure 1**. The final pragmatic OCTA bridge set comprised 134 participants.

#### Retinal outcome ascertainment and grading protocol

DR status was assigned at the eye level from protocol-based retinal imaging and clinical grading using the International Clinical Diabetic Retinopathy (ICDR) severity scale. The eye-level source sheet contained dedicated fields for grader 1, grader 2, the adjudicator, and the final consensus, documenting a two-grader adjudication workflow for every eye. Participant-level analyses used the final consensus grade. Grading was performed from the de-identified image-based records that did not display participant-level UACR, eGFR, or other systemic laboratory covariates during the image review, thereby reducing observer bias in eye-level outcome assignment. The final categories were no DR, mild non-proliferative DR, moderate non-proliferative DR, severe non-proliferative DR, and proliferative DR. The DME status and CI-DME were recorded separately, and vision-threatening diabetic retinopathy (VTDR) was defined as severe non-proliferative DR, proliferative DR, or CI-DME. Ungradable eyes were not eligible for participant-level outcome analysis. To align the clinical cohort with the participant-level NHANES inference and avoid within-participant correlation in the primary models, one analysis eye per participant was selected using a prespecified hierarchy: worse DR, then better OCTA quality if the DR severity was tied, and then the right eye. In tied cases, prioritizing the higher-quality OCTA scan could preferentially retain the less artifact-prone and potentially slightly less ischemic eye. In such a case, this choice would be expected to bias the UACR-OCTA associations conservatively toward the null rather than away from it. This possibility was examined in the bilateral eye-level generalized estimating equation (GEE) sensitivity analyses (16).

#### OCTA acquisition and image quality control

OCTA was acquired on a single platform, i.e., TowardPi 400k running TP-OCTA software version 2.8.1, with one fixed macular protocol of 6 mm × 6 mm and one fixed 150-degree ultrawidefield protocol, rather than mixed scan types within the same primary model. For each scan, the structured dataset recorded the device-reported signal score, the motion artifact, decentration, the segmentation quality, whether manual correction was required, and the primary metric exported from the device software. The final quality-pass status integrated the manufacturer-recommended quality criteria with a manual review of the motion artifact, centration, and the segmentation integrity. Widefield scans additionally tracked the gradable area, and macular scans tracked the foveal centration and layer-specific segmentation in the superficial, deep, and choriocapillaris slabs. Manual correction was allowed only when an otherwise interpretable scan could be salvaged, and only scans passing the final quality review were eligible for primary analysis. OCTA quality review and scan acceptance decisions were made from the de-identified scan-level files without reference to the participant-level laboratory sheet or the final clinical DR grade of the eye under review.

To limit multiplicity, the OCTA bridge prespecified one primary imaging endpoint and one main secondary endpoint. The primary endpoint was widefield total non-perfusion area from the 150-degree ultrawidefield scan because it most directly captured the ischemic phenotype hypothesized to parallel the NHANES albuminuria–DR signal. This endpoint was defined as the device-exported total capillary non-perfusion area in square millimeters generated by the TowardPi version 2.8.1 analysis module after quality control: manual region tracing or hand-drawn delineation of non-perfusion was not used. The TP-OCTA analysis module applies manufacturer-implemented segmentation and flow/non-perfusion quantification procedures. Detailed thresholding rules for non-perfusion area detection are proprietary and may differ from those implemented in other OCTA platforms, such as Optovue or Zeiss. The main secondary endpoint was macular superficial capillary plexus parafoveal perfusion density from the 6 × 6 scan. Other device-exported macular, deep plexus, and structural metrics were treated as descriptive or supplementary rather than co-primary outcomes.

#### Statistical analysis

The clinical replication outcomes were moderate-or-worse DR and VTDR in the prespecified analysis eye, with ordered DR rank used as a supportive ordinal outcome. The renal exposure of interest was UACR, measured directly in milligrams per gram from a single random spot urine specimen, thereby matching the spot urine framework used in the NHANES. The serum creatinine and companion chemistry measures were obtained from the same institutional clinical laboratory and were abstracted directly from the laboratory information system, reducing within-cohort assay heterogeneity by avoiding cross-laboratory calibration. Because the de-identified source workbook did not retain external proficiency program fields, we did not claim formal cross-laboratory recalibration. Instead, comparability across study parts relied on the shared spot urine ACR design and the creatinine-based eGFR framework. eGFR used the laboratory-reported value when available; otherwise, it was recalculated with the 2021 CKD-EPI creatinine equation. UACR was modeled primarily on the base-2 logarithmic scale; therefore, estimates represent the effect per doubling and secondarily in clinical categories of <30, 30–299, and 300 mg/g or higher. The continuous log2-transformed UACR models were designated *a priori* as primary because they use the full sample and are less vulnerable to sparse data instability in the highest UACR stratum. Category models were reserved for exploratory clinical illustration. The prespecified covariates were age, sex, diabetes duration, HbA1c, systolic blood pressure, BMI, and eGFR. Unlike in NHANES, the diabetes duration was directly and systematically recorded for all enrolled participants and was therefore included in all adjusted part 2 models. Systolic blood pressure was represented by the mean of the available clinic readings. Directly measured low-density lipoprotein cholesterol and triglycerides were complete in the source cohort and entered only in prespecified lipid-adjusted sensitivity analyses.

Primary part 2 analyses followed the inferential sequence of the study design. Firstly, the 168-participant clinical replication set was analyzed with multivariable logistic regression for moderate-or-worse DR and VTDR and proportional odds logistic regression for ordered DR severity. Secondly, the nested OCTA bridge set was analyzed with multivariable linear regression for the prespecified widefield primary endpoint and the main secondary macular endpoint. The OCTA outcomes were standardized within the analytic set before modeling so that the beta coefficients represented standard deviation (SD) differences and could be compared across imaging endpoints. Parallel raw-scale models using the original units were fit for clinical interpretation and are reported narratively. Category-based UACR models, lipid-adjusted analyses, approximate *E*-value analyses, and all other sensitivity analyses were prespecified as supportive rather than alternative primary analyses.

The single-center cohort was a pragmatic consecutive sample rather than a formally powered trial; therefore, we did not perform *post-hoc* power calculations. Precision was conveyed with 95% confidence intervals. As a design-sensitivity descriptor, the pragmatic OCTA set corresponded to a minimum detectable standardized UACR effect of approximately 0.06–0.07 SD for the two prespecified OCTA endpoints at 80% power with a two-sided alpha of 0.05 under the observed covariate structure. Because sparse events in the highest UACR category could destabilize ordinary categorical clinical models, category-based clinical analyses were re-estimated with Firth’s penalized logistic regression and interpreted as exploratory clinical illustrations rather than primary inference. To evaluate the selection introduced by OCTA eligibility and quality exclusions, we first compared the clinically eligible participants who did and did not enter the pragmatic OCTA set and then fit the inverse-probability-of-selection-weighted OCTA sensitivity models. Because some exclusions, such as recent retinal treatment or major ocular exclusion, created structural non-overlap, these weighted models were interpreted as bias sensitivity analyses rather than full transportability corrections. Among the 168 clinically eligible participants, a parsimonious logistic selection model based on age, sex, log2-transformed UACR, eGFR, ordered DR rank, and CI-DME generated stabilized weights truncated at the first and 99th percentiles and applied to the covariate-adjusted OCTA models. As a complementary sensitivity analysis for unmeasured confounding, we calculated an approximate *E*-value for the prespecified widefield primary endpoint. The *E*-value represents the minimum risk ratio scale strength an unmeasured confounder would need with both the exposure contrast and the outcome, conditional on the measured covariates, in order to explain away the observed association. For interpretability, the *E*-value was anchored to a clinically meaningful 30- to 300-mg/g UACR contrast by translating the standardized effect estimate to the risk ratio scale. Because this transformation is approximate for continuous outcomes, it was treated as supportive rather than primary. Routine diagnostic review included variance inflation factors, Hosmer–Lemeshow calibration for logistic models, and residual and influence review for the OCTA linear models. As the clinical datasets were intentionally assembled as complete case analysis files with all prespecified covariates present, no imputation was performed. No formal multiple comparison correction was used to define the primary inferential rule. Instead, inference emphasized the prespecified hierarchy of primary, main secondary, and supportive endpoints together with the effect sizes and 95% confidence intervals. For transparency, we additionally report whether the six principal UACR associations exceeded a conservative Bonferroni reference threshold of *α* = 0.0083. Sensitivity analyses repeated the OCTA models in the strict widefield quality set with 126 participants, the strict macular quality set with 123, the CI-DME-excluded set with 125, and the short laboratory window set of 39 participants tested within 7 days of OCTA using the same covariate-adjusted framework. Bilateral eye-level models were also fit with GEEs using an exchangeable working correlation and robust standard errors. All statistical analyses were performed using R software, version 4.3.1 (R Foundation for Statistical Computing, Vienna, Austria). Penalized logistic regression was performed using the *logistf* package, and GEEs were modeled using the *geepack* package. All tests were two-sided, and exact *p*-values are reported when *p* ≥ 0.001 and smaller values as *p* < 0.001.

## Results

### Overview of the analytic cohorts

The final analytic sequence mirrored the staged design. The NHANES discovery cohort included 964 participants, of whom 949 contributed complete case adjusted models. The single-center enrolled source cohort included 180 participants, the clinical replication set 168, and the nested pragmatic OCTA bridge set 134. The NHANES complete case models included 293 AnyDR events and 94 SevereDR events. The prespecified OCTA sensitivity subsets included a strict widefield quality set with 126 participants, a strict macular quality set with 123, and a CI-DME-excluded set with 125. Because OCTA eligibility excluded eyes after recent PRP or anti-VEGF, vitrectomy, dense media opacity, or non-interpretable scans, the pragmatic bridge set should be interpreted as a snapshot of the pre-destructive intervention retinal microvascular state rather than the full spectrum of treated late-stage disease.

#### Part 1: NHANES discovery cohort

##### Participant characteristics

In the NHANES analytic cohort of 964 participants, retinal outcomes were available for all participants and missingness in the adjusted model covariates was minimal. Only 15 participants were excluded from the multivariable models due to overlapping missingness in the eGFR, systolic blood pressure, or HbA1c: the corresponding missing counts were six, six, and four. After application of the 4-year MEC weights, these estimates represent US adults with diabetes aged 40 years or older who attended the MEC and had gradable retinal photographs. The survey-weighted mean age was 60.3 ± 0.5 years, 50.2% were men, and the race and ethnicity distribution was 65.6% non-Hispanic White, 13.0% Hispanic, 16.3% non-Hispanic Black, and 5.2% other or multiracial. The original five-level RIDRETH1 distribution is provided in **Supplementary Table 1**. The survey-weighted prevalence was 27.1% ± 1.8% for AnyDR and was 7.7% ± 1.0% for SevereDR. Other weighted baseline characteristics included a mean BMI of 32.8 ± 0.3 kg/m^2^, systolic blood pressure of 131.5 ± 1.0 mmHg, HbA1c of 7.16% ± 0.08%, and eGFR of 78.7 ± 0.9 ml/min per 1.73 m^2^. **Table 1** provides the full details.

**Table 1:** Baseline characteristics of the National Health and Nutrition Examination Survey (NHANES) analytic cohort, 964 adults aged ≥40 years with diabetes and gradable fundus photographs, 2005–2008.

Characteristic	Overall (survey-weighted)	Unweighted *n*	Missing *n* (%)
Participants (analytic sample)	–	964	–
Any diabetic retinopathy (AnyDR), prevalence	27.1% ± 1.8%	964	0 (0.00)
Severe diabetic retinopathy (SevereDR), prevalence	7.7% ± 1.0%	964	0 (0.00)
Age (years)	60.3 ± 0.5	964	0 (0.00)
BMI (kg/m^2^)	32.8 ± 0.3	964	0 (0.00)
Systolic BP (mmHg)	131.5 ± 1.0	964	6 (0.62)
HbA1c (%)	7.16 ± 0.08	964	4 (0.41)
eGFR (ml/min per 1.73 m^2^)	78.7 ± 0.9	964	6 (0.62)
ln(ACR)	2.86 ± 0.05	964	0 (0.00)
Sex	–	964	0 (0.00)
Male	50.2%	491	0 (0.00)
Female	49.8%	473	0 (0.00)
Race/ethnicity (race4)	–	964	0 (0.00)
Non-Hispanic White	65.6%	392	0 (0.00)
Hispanic	13.0%	273	0 (0.00)
Non-Hispanic Black	16.3%	272	0 (0.00)
Other/multiracial	5.2%	27	0 (0.00)

Continuous variables are presented as survey-weighted mean ± standard errors. Categorical variables are presented as survey-weighted percentages only. Unweighted counts are shown separately. Missing *n* (%) are unweighted counts.

*DR*, diabetic retinopathy; *eGFR*, estimated glomerular filtration rate; *ACR*, albumin-to-creatinine ratio.

##### Independent association of ACR with AnyDR and SevereDR

In the survey-weighted multivariable models adjusted for age, sex, race and ethnicity, BMI, systolic blood pressure, HbA1c, and eGFR, a higher ACR was independently associated with both retinal outcomes, whereas eGFR was not. Each doubling of ACR was associated with higher odds of AnyDR (adjusted OR = 1.18, 95%CI = 1.09–1.27, *p* < 0.001) and SevereDR (adjusted OR = 1.30, 95%CI = 1.18–1.43, *p* < 0.001) (**Table 2**). In contrast, eGFR showed no independent association with AnyDR (OR = 0.99, 95%CI = 0.98–1.00, *p* = 0.190) or SevereDR (OR = 0.99, 95%CI = 0.97–1.00, *p* = 0.081). HbA1c remained a strong independent covariate in both models, and non-Hispanic Black participants had higher adjusted odds of both AnyDR and SevereDR than non-Hispanic White participants. Reanalysis with the original five-level NHANES race and ethnicity variable produced materially similar coefficients for both ACR and eGFR (**Supplementary Tables 2**, **3**).

**Table 2:** Survey-weighted associations of albumin-to-creatinine ratio (ACR) and estimated glomerular filtration rate (eGFR) with AnyDR and SevereDR in the National Health and Nutrition Examination Survey (NHANES).

Variable	AnyDR OR	95%CI	*p*-value	SevereDR OR	95%CI	*p*-value
Model A: ACR as log2(ACR) (per doubling)						
log2(ACR) (per doubling)	1.18	1.09–1.27	<0.001	1.30	1.18–1.43	<0.001
eGFR (per 1 ml/min per 1.73 m^2^)	0.99	0.98–1.00	0.190	0.99	0.97–1.00	0.081
HbA1c (per 1%)	1.53	1.32–1.78	<0.001	1.45	1.25–1.67	<0.001
Non-Hispanic Black *vs*. non-Hispanic White	1.77	1.06–2.97	0.040	2.28	1.26–4.12	0.010
Model B: ACR as clinical categories						
ACR 30–299 mg/g *vs*. <30 mg/g	1.14	0.82–1.58	0.440	1.68	0.96–2.95	0.085
ACR ≥300 mg/g *vs*. <30 mg/g	3.85	2.02–7.34	<0.001	3.96	1.98–7.92	<0.001
Trend per category increase	1.58	1.20–2.08	0.004	–	–	–

The complete case sample size for primary models was 949, including 293 AnyDR events and 94 SevereDR events. Models included log2-transformed ACR, eGFR, age, sex, race/ethnicity, body mass index, systolic blood pressure, and HbA1c. Diabetes duration was not included because a uniformly measured duration variable was not available across the full NHANES retinal photography cohort. Log2-transformed ACR denotes the base-2 logarithm of ACR. A 1-unit increase corresponds to a doubling of ACR. For Model B, clinical ACR categories replaced the log2-transformed ACR and the same covariates were retained. These category results are supportive clinical illustrations.

##### Dose–response pattern

Supportive category-based models localized the excess retinal risk to the highest albuminuria stratum. Relative to ACR below 30 mg/g, an ACR of 300 mg/g or higher was associated with higher odds of AnyDR (adjusted OR = 3.85, 95%CI = 2.02–7.34) and SevereDR (adjusted OR = 3.96, 95%CI = 1.98–7.92), whereas the 30- to 299-mg/g stratum showed weaker and less precise associations. Ordered trend across the ACR categories remained significant for AnyDR, with an OR of 1.58 per category increase (95%CI = 1.20–2.08, *p* = 0.004). Restricted cubic splines for ACR showed no strong evidence of nonlinearity, with a *p*-value for nonlinearity of 0.098, supporting the prespecified log2-transformed ACR specification as the primary inferential model. A parallel exploratory eGFR spline is provided descriptively in **Supplementary Figure 2**. Exploratory piecewise modeling suggested a flatter association below approximately 55.3 mg/g, with an OR of 1.03 per doubling (95%CI = 0.91–1.17), and a steeper association above that level (OR = 1.44, 95%CI = 1.24–1.66). Because formal nonlinearity was not demonstrated, these analyses were interpreted as descriptive rather than alternative primary models (**Supplementary Figure 3**, **Supplementary Table 5**).

Overall, the NHANES discovery analysis showed a consistent pattern. Albuminuria was associated with retinopathy at the population level, the association strengthened for the more advanced retinal endpoint, and the findings were robust to alternative race and ethnicity coding and descriptive shape analyses. In contrast, the eGFR remained null across the same checks.

#### Part 2: Single-center clinical replication and OCTA bridge

##### Cohort derivation and baseline characteristics

The single-center ophthalmic specialty clinic source cohort comprised 180 consecutively enrolled adults with type 2 diabetes, and all 180 already had UACR, serum creatinine and eGFR, HbA1c, systolic blood pressure, and protocol-based participant-level DR grading recorded in the structured source workbook. Thus, part 2 derivation was not driven by missing core kidney or retinal variables. There were 12 participants who did not meet the prespecified laboratory anchoring criterion as the nearest laboratory panel was obtained more than 30 days from OCTA, ranging from 32 to 57 days, leaving 168 participants in the clinical replication set. The median laboratory-to-OCTA interval in this set was 12 days, with an interquartile range of 6–19 days. After prespecified single-eye selection and protocol-specified ocular, treatment-related, and imaging exclusions, 134 participants entered the nested pragmatic OCTA bridge set. The nested sensitivity sets included a strict widefield quality set with 126 participants, a strict macular quality set with 123, a CI-DME-excluded set with 125, and a short laboratory window subset of 39 participants tested within 7 days of OCTA (**Figure 1B**).

The baseline characteristics are summarized in **Table 3**. In the 168-participant clinical replication set, 68 participants had UACR below 30 mg/g, 62 had UACR 30–299 mg/g, and 38 had UACR 300 mg/g or higher. The mean age was 56.0 ± 10.0 years, 47.6% were men, the mean diabetes duration was 8.8 ± 4.7 years, the mean HbA1c was 8.1% ± 1.1%, the mean eGFR was 89.0 ± 16.7 ml/min per 1.73 m^2^, and the median UACR was 74.5 mg/g, with an interquartile range of 18.0–214.5. Moderate-or-worse DR, VTDR, proliferative DR, and CI-DME were present in 54.8%, 34.5%, 19.6%, and 11.9% of the participants, respectively. In the nested OCTA set, the corresponding UACR stratum counts were 60, 49, and 25 participants. The mean widefield total non-perfusion area was 6.5 ± 4.7 mm^2^, and the mean macular superficial capillary plexus parafoveal perfusion density was 49.1% ± 2.6%. Compared with the NHANES, which represented a broader diabetes population with 27.1% AnyDR, the single-center cohort was intentionally enriched for higher-acuity ophthalmic disease, with 54.8% moderate-or-worse DR and 34.5% VTDR. Part 2 therefore examined whether the same renal–retinal signal persists in a more severe specialty clinic case mix rather than attempting prevalence matching across cohorts.

**Table 3:** Cohort characteristics across single-center analysis sets.

Characteristic	All enrolled	Clinical replication	Pragmatic OCTA	Strict widefield QC
*N*	180	168	134	126
Age (years)	56.0 ± 9.9	56.0 ± 10.0	54.5 ± 9.5	54.3 ± 9.3
Male sex	88 (48.9%)	80 (47.6%)	64 (47.8%)	60 (47.6%)
Diabetes duration (years)	8.7 ± 4.7	8.8 ± 4.7	7.8 ± 4.4	7.7 ± 4.4
BMI (kg/m^2^)	26.0 ± 3.7	26.0 ± 3.7	26.1 ± 3.7	26.2 ± 3.6
Systolic blood pressure (mmHg)	131.9 ± 10.7	131.8 ± 10.6	130.7 ± 10.7	130.6 ± 10.8
HbA1c (%)	8.1 ± 1.1	8.1 ± 1.1	7.9 ± 1.0	7.9 ± 1.0
eGFR (ml/min per 1.73 m^2^)	89.4 ± 16.9	89.0 ± 16.7	92.1 ± 14.9	92.5 ± 14.7
UACR (mg/g)	74.5 (18.0–214.5)	74.5 (18.0–214.5)	57.0 (16.2–163.5)	50.5 (16.0–161.5)
UACR ≥300 mg/g	41 (22.8%)	38 (22.6%)	25 (18.7%)	22 (17.5%)
Moderate-or-worse DR	98 (54.4%)	92 (54.8%)	63 (47.0%)	57 (45.2%)
VTDR	62 (34.4%)	58 (34.5%)	29 (21.6%)	24 (19.0%)
Proliferative DR	36 (20.0%)	33 (19.6%)	10 (7.5%)	8 (6.3%)
Center-involving DME	22 (12.2%)	20 (11.9%)	9 (6.7%)	7 (5.6%)
Widefield total non-perfusion area (mm2)	7.4 ± 5.3	7.5 ± 5.3	6.5 ± 4.7	6.2 ± 4.4
Macular SCP parafoveal perfusion density (%)	48.5 ± 2.9	48.5 ± 2.9	49.1 ± 2.6	49.2 ± 2.6

Values are the mean ± SD, median with interquartile range, or *n* (%), as appropriate. All 180 enrolled participants had urine UACR, serum creatinine and eGFR, HbA1c, systolic blood pressure, and participant-level DR grading available. The reduction to 168 reflected only the prespecified requirement that the closest laboratory panel fall within 30 days of OCTA.

*OCTA*, optical coherence tomography angiography; *HbA1c*, glycated hemoglobin; *eGFR*, estimated glomerular filtration rate; *UACR*, urine albumin-to-creatinine ratio; *DR*, diabetic retinopathy; *VTDR*, vision-threatening diabetic retinopathy; *DME*, diabetic macular edema; *SCP*, superficial capillary plexus.

Compared with the 34 clinically eligible participants excluded from the pragmatic OCTA set, the 134 included participants were younger and clinically milder. The excluded participants had longer diabetes duration, 12.4 ± 4.2 *vs*. 7.8 ± 4.4 years; higher UACR, with a median of 146.5 mg/g and an interquartile range of 73.3–487.0 *vs*. 57.0 mg/g and an interquartile range of 16.3–163.5; lower eGFR, 76.7 ± 17.9 *vs*. 92.1 ± 14.9 ml/min per 1.73 m^2^; and a substantially more advanced DR, with VTDR in 85.3% *vs*. 21.6%. This pattern was consistent with more severe eyes being disproportionately excluded due to recent PRP or anti-VEGF therapy, prior vitrectomy, media opacity, or inadequate OCTA image quality (**Supplementary Table 12C**).

##### Clinical replication of the renal–retinal association

**Figure 2:**
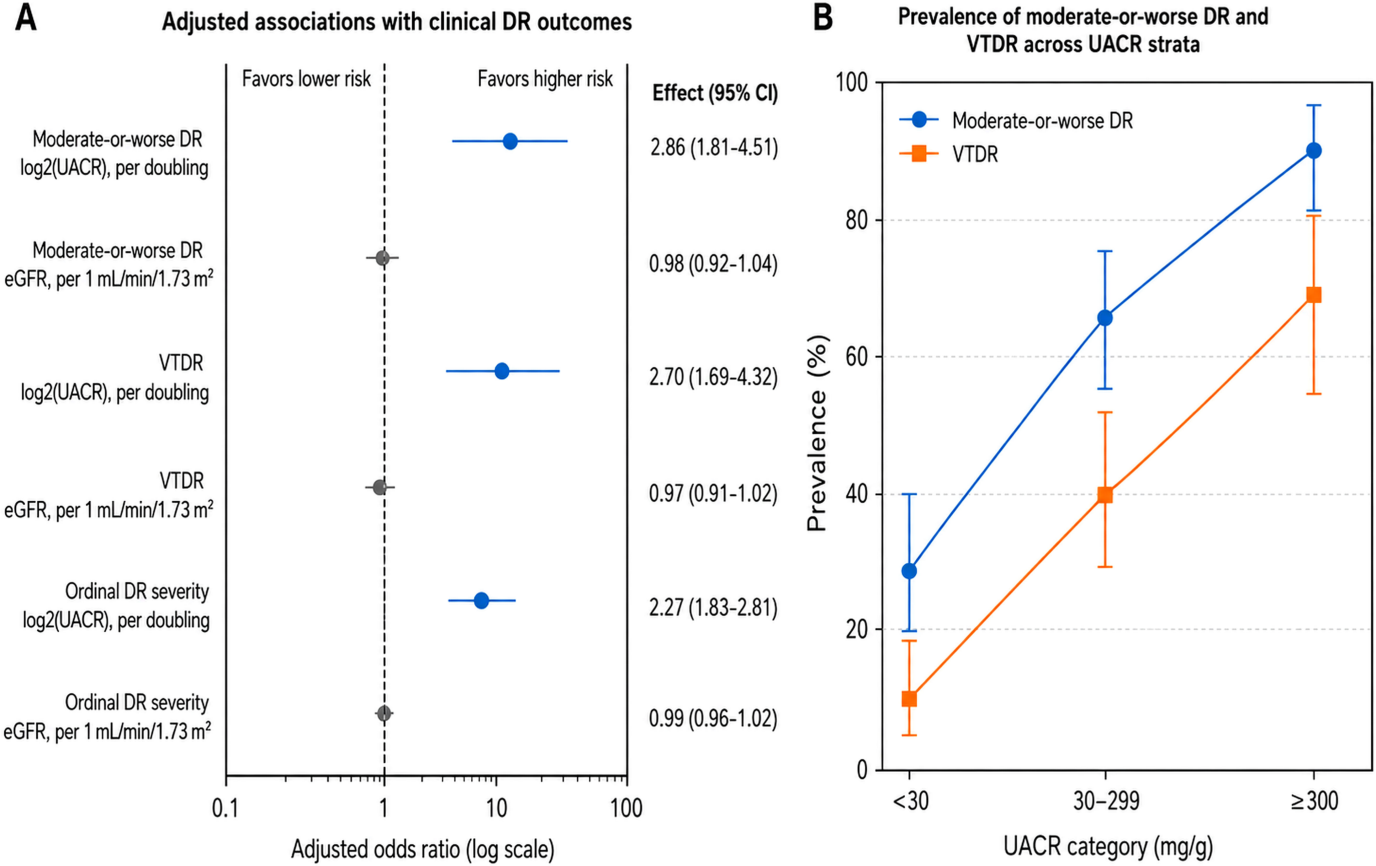
Clinical replication of the UACR–DR association. **(A)** shows adjusted odds ratios and 95% confidence intervals from the prespecified primary continuous-exposure models for moderate-or-worse DR, VTDR, and ordinal DR severity in the single-center cohort. Models were adjusted for age, sex, diabetes duration, HbA1c, systolic blood pressure, body mass index, and eGFR when UACR was the exposure; UACR was included when eGFR was evaluated. log2-transformed UACR is interpreted per doubling of UACR. **(B)** shows observed prevalence and 95% confidence intervals for moderate-or-worse DR and VTDR across clinical UACR categories below 30 mg/g, 30 to 299 mg/g, and 300 mg/g or higher.

The prespecified continuous log2-transformed UACR models, designated *a priori* as the primary inferential basis for part 2, reproduced both the direction and the specificity of the NHANES signal despite the markedly higher-risk case mix. After multivariable adjustment, each doubling of UACR was associated with higher odds of moderate-or-worse DR (adjusted OR = 2.86, 95%CI = 1.81–4.51, *p* < 0.001) and VTDR (adjusted OR = 2.70, 95%CI = 1.69–4.32, *p* < 0.001). eGFR, again, was not independently associated with either endpoint (**Table 4**, **Figure 2A**). Supportive proportional odds models across the full ordered DR scale showed the same pattern for UACR (OR = 2.27, 95%CI = 1.83–2.81, *p* < 0.001), with no independent association for eGFR (OR = 0.99, 95%CI = 0.96–1.02, *p* = 0.331). This null eGFR signal persisted despite the remaining variation in kidney function within the clinical replication set, where the mean eGFR was 89.0 ± 16.7 ml/min per 1.73 m^2^ and the range was 45–125. However, as only nine participants had eGFR below 60 ml/min per 1.73 m^2^, the cohort had limited information in the reduced eGFR range. These null eGFR estimates should therefore be interpreted as an absence of detectable independent association within a largely preserved kidney function distribution rather than as evidence that eGFR is unrelated to DR in populations with more advanced chronic kidney disease (CKD). On the absolute scale, the prevalence of moderate-or-worse DR increased from 27.9% to 89.5% and VTDR from 10.3% to 68.4% across the lowest to the highest UACR strata.

**Table 4:** Primary continuous models for clinical replication endpoints in the single-center cohort.

Outcome	Exposure	Adjusted effect (95%CI)	*p*-value	*N*
Moderate-or-worse DR	log2(UACR), per doubling	2.86 (1.81–4.51)	<0.001	168
Moderate-or-worse DR	eGFR, per 1 ml/min per 1.73 m^2^	0.98 (0.92–1.04)	0.421	168
VTDR	log2(UACR), per doubling	2.70 (1.69–4.32)	<0.001	168
VTDR	eGFR, per 1 ml/min per 1.73 m^2^	0.97 (0.91–1.02)	0.235	168
Ordinal DR severity (supportive)	log2(UACR), per doubling	2.27 (1.83–2.81)	<0.001	168
Ordinal DR severity (supportive)	eGFR, per 1 ml/min per 1.73 m^2^	0.99 (0.96–1.02)	0.331	168

Models were adjusted for age, sex, diabetes duration, HbA1c, systolic blood pressure, body mass index, and eGFR when UACR was the exposure. UACR was included when eGFR was evaluated. Effect estimates are not intended for direct quantitative comparison with the National Health and Nutrition Examination Survey (NHANES) estimates as diabetes duration was available and adjusted for in part 2, but not in the primary NHANES models. These continuous per-doubling models constituted the primary inferential basis for the single-center clinical replication analysis. Category-based UACR models are reported separately as exploratory clinical illustrations.

*DR*, diabetic retinopathy; *VTDR*, vision-threatening diabetic retinopathy; *UACR*, urine albumin-to-creatinine ratio; *eGFR*, estimated glomerular filtration rate.

Exploratory category-based analyses, interpreted chiefly through absolute risks rather than sparse stratum ORs, showed a steep clinical gradient across UACR strata. The observed prevalence of moderate-or-worse DR rose from 27.9% (19 of 68 participants) in those with UACR below 30 mg/g to 62.9% (39 of 62) in the 30–299 mg/g group and 89.5% (34 of 38) in the 300 mg/g or higher group. The corresponding VTDR prevalence rates were 10.3% (7 of 68), 40.3% (25 of 62), and 68.4% (26 of 38) (**Figure 2B**). Once UACR reached 300 mg/g or higher, the absolute prevalence of moderate-or-worse DR was higher by 61.6 percentage points and the absolute prevalence of VTDR by 58.1 percentage points relative to UACR below 30 mg/g. Exploratory category-based Firth models preserved the same monotonic ordering across strata, but with the highest UACR stratum having very high event rates; therefore, the penalized ORs were numerically large and imprecise, as expected under near separation. Accordingly, the absolute prevalence gradient is more clinically interpretable than any single extreme OR estimate in the highest UACR stratum (**Supplementary Table 10**).

##### OCTA bridge to ischemic retinal phenotype

**Figure 3:**
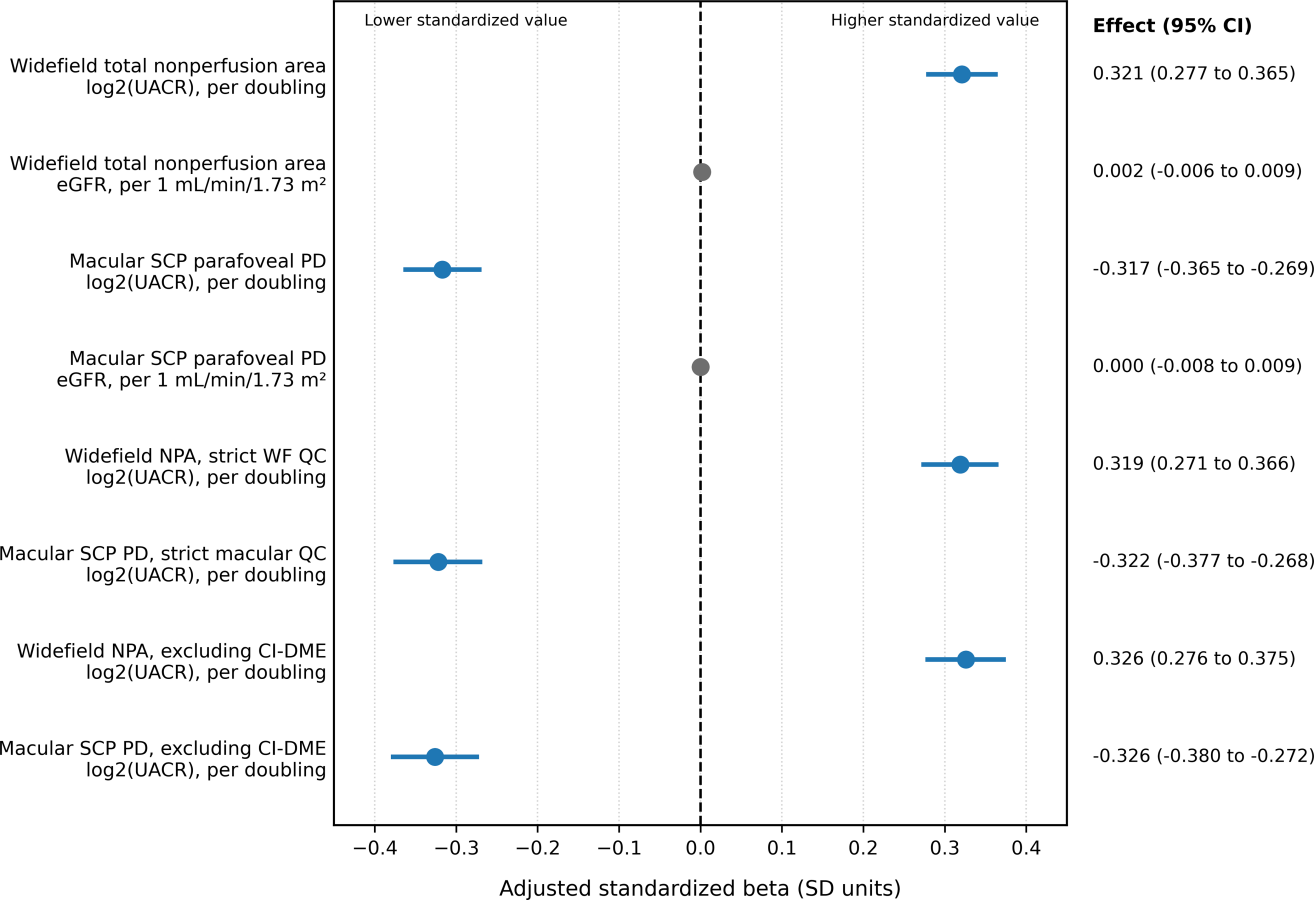
Adjusted associations with ischemic retinal OCTA phenotype. Points and horizontal lines denote adjusted standardized beta coefficients and 95% confidence intervals from the prespecified primary continuous-exposure OCTA models for widefield total nonperfusion area and macular superficial capillary plexus parafoveal perfusion density. Sensitivity analyses additionally show the strict widefield quality-pass set, strict macular quality-pass set, and the pragmatic set after exclusion of CI-DME. Positive beta coefficients for widefield nonperfusion indicate greater ischemic burden, whereas negative beta coefficients for macular perfusion density indicate lower perfusion. OCTA metrics were generated using the TowardPi 400k platform and TP-OCTA software version 2.8.1; device-exported nonperfusion-area metrics should therefore be interpreted as TowardPi/TP-OCTA-derived measurements.

Within the nested pragmatic OCTA bridge set, the renal–retinal association aligned with an ischemic imaging phenotype. A higher log2-transformed UACR was independently associated with greater widefield total non-perfusion area, the prespecified primary imaging endpoint, with a standardized beta of 0.321 per doubling (95%CI = 0.277–0.365, *p* < 0.001). On the raw scale, this corresponded to an adjusted increase of 1.49 mm^2^ per doubling of UACR (95%CI = 1.28–1.69). Given the cohort mean widefield non-perfusion burden of 6.5 ± 4.7 mm^2^, this increment represents roughly 23% of the average ischemic burden per doubling and is clinically meaningful. A higher UACR was also associated with a lower macular superficial capillary plexus parafoveal perfusion density, the main secondary OCTA endpoint, with a standardized beta of −0.317 (95%CI = −0.365 to −0.269, *p* < 0.001), corresponding to an adjusted raw scale change of −0.82 percentage points (95%CI = −0.95 to −0.70). Consistent with the clinical replication analysis, eGFR was not independently associated with either widefield non-perfusion (*β* = 0.002, 95%CI = −0.006 to 0.009, *p* = 0.687) or macular perfusion density (*β* = 0.000, 95%CI = −0.008 to 0.009, *p* = 0.950) (**Table 5**, **Figure 3**).

**Table 5:** Primary continuous models for optical coherence tomography angiography (OCTA) bridge endpoints in the single-center cohort.

Outcome	Exposure	Adjusted beta, SD units (95%CI)	*p*-value	*N*
Widefield total non-perfusion area (*z*)	log2(UACR), per doubling	0.321 (0.277–0.365)	<0.001	134
Widefield total non-perfusion area (*z*)	eGFR, per 1 ml/min per 1.73 m^2^	0.002 (−0.006 to 0.009)	0.687	134
Macular SCP parafoveal perfusion density (*z*)	log2(UACR), per doubling	−0.317 (−0.365 to −0.269)	<0.001	134
Macular SCP parafoveal perfusion density (*z*)	eGFR, per 1 ml/min per 1.73 m^2^	0.000 (−0.008 to 0.009)	0.950	134

The OCTA metrics were generated using the TowardPi 400k platform and TP-OCTA software version 2.8.1. Beta coefficients are standardized outcome differences per doubling of the UACR or per 1 ml/min per 1.73 m^2^ higher eGFR. Device-exported non-perfusion area metrics should be interpreted as TowardPi/TP-OCTA-derived measurements. Positive coefficients for widefield non-perfusion indicate more ischemia, whereas negative coefficients for macular perfusion density indicate lower perfusion. For clinical interpretability, the corresponding adjusted raw-scale change per doubling of the UACR was +1.49 mm^2^ for widefield non-perfusion and −0.82 percentage points for macular perfusion density. These continuous per-doubling models constituted the primary inferential basis for the OCTA bridge analysis. Category-based OCTA gradients are reported separately as supportive clinical illustrations.

*SCP*, superficial capillary plexus; *UACR*, urine albumin-to-creatinine ratio; *eGFR*, estimated glomerular filtration rate.

**Figure 4:**
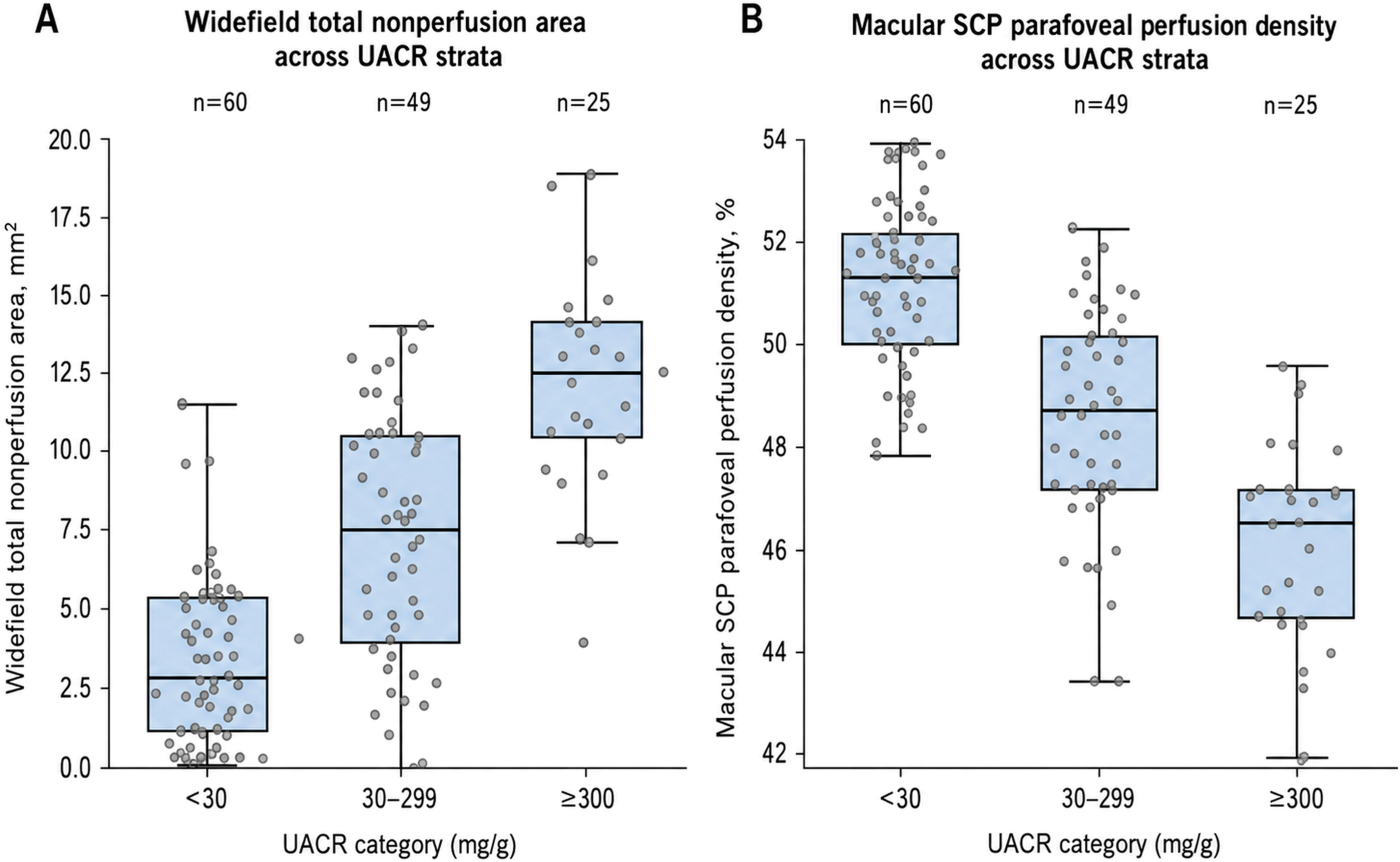
OCTA metrics across UACR strata. **(A)** shows widefield total nonperfusion area across clinical UACR categories, reported in mm². **(B)** shows macular superficial capillary plexus parafoveal perfusion density across clinical UACR categories, reported as a percentage. Boxplots show medians and interquartile ranges, whiskers indicate the 1.5 × interquartile range, and points indicate participant-level values. UACR categories were below 30 mg/g, 30 to 299 mg/g, and 300 mg/g or higher.

The exploratory descriptive and category-based OCTA analyses showed the same ordered gradient. The mean widefield total non-perfusion area increased from 3.4 ± 2.6 in participants with UACR below 30 mg/g to 7.2 ± 3.9 in those with UACR 30–299 mg/g and 12.3 ± 3.7 in those with UACR 300 mg/g or higher. Conversely, the mean macular perfusion density declined from 50.9 ± 1.6 to 48.4 ± 2.1 and 46.0 ± 1.9 across the same strata (**Figures 4A, B**; **Supplementary Tables 8**, **9**). **Figure 4A** also shows a broader upper tail in the highest UACR group, ranging from 4.04 to 20.13 mm^2^, which indicates substantial heterogeneity in ischemic burden within the heaviest albuminuria stratum. In the adjusted categorical OCTA models, relative to UACR below 30 mg/g, the 30–299 mg/g and 300 mg/g or higher strata were associated with standardized increases of 0.723 and 1.688 SD in widefield non-perfusion and standardized decreases of 0.860 and 1.645 SD in macular perfusion density, respectively (**Supplementary Table 11**). These category gradients were directionally consistent with the prespecified per-doubling continuous models, but were interpreted only as supportive clinical illustrations.

##### Sensitivity analyses

Prespecified sensitivity analyses addressed the scan quality thresholds, CI-DME-related segmentation distortion, short laboratory-to-OCTA timing, measured differential inclusion into the pragmatic OCTA set, additional lipid adjustment in the single-center cohort, and the overall model robustness. The association between UACR and widefield non-perfusion remained essentially unchanged in the strict widefield quality pass set of 126 participants (standardized *β* = 0.319, 95%CI = 0.271–0.366, *p* < 0.001), and the association with lower macular perfusion density remained stable in the strict macular quality pass set of 123 participants (standardized *β* = −0.322, 95%CI = −0.377 to −0.268, *p* < 0.001) (**Figure 3**; **Supplementary Table 12A**). Excluding CI-DME in 125 participants likewise did not materially change the estimates for widefield non-perfusion (*β* = 0.326, 95%CI = 0.276–0.375, *p* < 0.001) or macular perfusion density (*β* = −0.326, 95%CI = −0.380 to −0.272, *p* < 0.001) (**Supplementary Table 12A**). Restricting the OCTA bridge analysis to the 39 participants whose laboratory testing occurred within 7 days of OCTA attenuated precision, but preserved the same directional association for widefield non-perfusion (*β* = 0.225, 95%CI = 0.090–0.361, *p* = 0.002) and macular perfusion density (*β* = −0.290, 95%CI = −0.400 to −0.181, *p* < 0.001) (**Supplementary Table 12A**). Additional adjustment for directly measured low-density lipoprotein cholesterol and triglycerides did not materially change the clinical or OCTA results, with the UACR signal and null eGFR findings essentially unchanged across endpoints (**Supplementary Table 12E**). The selection-weighted sensitivity analyses that reweighted the 134 included participants toward the full 168-participant clinically eligible set were nearly identical to the primary OCTA models. The adjusted association of UACR remained 0.327 SD (95%CI = 0.282–0.372, *p* < 0.001) for widefield non-perfusion and −0.320 SD (95%CI = −0.367 to −0.273, *p* < 0.001) for macular perfusion density, while the eGFR remained null for both endpoints (**Supplementary Table 12D**). For the prespecified widefield primary endpoint, an approximate *E*-value analysis anchored to a clinically interpretable 30- to 300-mg/g UACR contrast yielded an *E*-value of 4.72 for the point estimate and 4.05 for the lower 95% confidence limit. This implies that an unmeasured confounder would need roughly fourfold associations with both the exposure contrast and higher widefield non-perfusion, beyond the measured covariates, to move the confidence interval to the null (**Supplementary Table 13**). Routine diagnostic review also supported model robustness. The variance inflation factors were all 2.41 or lower in the clinical set and 1.96 or lower in the pragmatic OCTA set, and the Hosmer–Lemeshow calibration was acceptable for the two clinical logistic models, with *p*-values of 0.865 and 0.953. The standardized OCTA residuals were approximately normal, and no externally studentized residual exceeded 2.46. The near-identical effect estimates across these filtered and selection-weighted analyses reduce concern that the primary OCTA findings were driven by CI-DME-related segmentation distortion, measured differential inclusion, short-term laboratory timing mismatch, or a small number of extreme observations. As a robustness check rather than the primary inferential criterion, all six principal UACR associations in the integrated study also exceeded a conservative Bonferroni reference threshold of *α* = 0.0083.

Allowing both eyes to contribute also did not alter inference. In the clinical GEE set, which included 336 eyes from 168 participants, the adjusted OR per doubling of UACR was 2.24 (95%CI = 1.80–2.79) for moderate-or-worse DR and was 2.05 (95%CI = 1.67–2.53) for VTDR. In the OCTA GEE set, which included 246 eyes from 134 participants, the corresponding standardized beta coefficients were 0.330 (95%CI = 0.292–0.368) for widefield total non-perfusion area and was −0.296 (95%CI = −0.338 to −0.254) for macular superficial capillary plexus parafoveal perfusion density (**Supplementary Table 12B**). Across all models in which eGFR was entered simultaneously with UACR, including the primary clinical and OCTA models, the quality-filtered and CI-DME-excluded OCTA analyses, short-window and selection-weighted models, bilateral eye GEE models, and lipid-adjusted sensitivity analyses, no independent association between eGFR and any retinal endpoint was detected, with all *p*-values greater than 0.05 (**Supplementary Table 12**). Because the excluded participants were older and had longer diabetes duration, lower eGFR, higher UACR, and substantially more advanced DR (**Supplementary Table 12C**), these OCTA ischemic findings should be interpreted chiefly as describing the pre-destructive intervention retinal microvascular state in eyes that remained gradable and had not recently undergone PRP, anti-VEGF therapy, or vitrectomy rather than eyes already altered by treatment or dense media opacity.

## Discussion

In this staged discovery replication analysis, albuminuria emerged as the renal measure most consistently associated with diabetic retinal disease. Across both a nationally representative NHANES cohort and an independent higher-acuity ophthalmic cohort, higher albuminuria, measured as ACR in the NHANES and UACR in the clinical cohort, was independently associated with more severe DR, whereas eGFR was not. In the nested OCTA bridge analysis, a higher UACR also corresponded to greater retinal non-perfusion and lower macular perfusion density, extending the renal–retinal association from clinical grading to an imaging-defined ischemic phenotype. Together, these findings suggest that albuminuria captures a dimension of systemic microvascular injury more closely aligned with retinal disease burden than filtration-based kidney function.

Prior studies have reported inconsistent associations of albuminuria and eGFR with DR, in part because renal exposures were not always evaluated simultaneously, cohorts differed substantially in baseline risk, and the retinal outcomes were often limited to conventional severity categories. Our design addresses these limitations by jointly modeling albuminuria and eGFR, examining the association in a population-based discovery cohort and an independent clinical replication cohort, and then linking the clinical finding to the OCTA-derived ischemic burden. The consistency of the signal across these analytic levels strengthens the inference that albumin leakage, rather than reduced filtration per se, is the renal correlate most closely tied to retinal microvascular injury (17, 18).

A biologically coherent explanation supports this pattern. The retinal capillary bed and the glomerular filtration barrier share specialized endothelia, basement membranes, and susceptibility to diabetes-related endothelial glycocalyx injury. In the kidney, this injury is expressed clinically as albumin leakage across a disrupted barrier; in the retina, it may manifest as capillary closure, impaired perfusion, and progressive ischemia. Albuminuria therefore may register endothelial barrier damage at an earlier stage than creatinine-based eGFR, which summarizes whole kidney filtration function rather than focal microvascular leak. In contrast, eGFR is an integrative functional measure influenced by factors beyond microvascular barrier injury, including age, muscle mass, nephron reserve, and intraglomerular hemodynamics. In the earlier stages of diabetes, hyperfiltration may preserve or even elevate the eGFR despite evolving retinal disease, making albuminuria a more sensitive tracker of early retinal microvascular compromise after joint adjustment (19).

The null findings for eGFR also require cautious interpretation. In the single-center clinical cohort, kidney function was largely preserved and only nine participants had eGFR below 60 ml/min per 1.73 m^2^, which limited the statistical power to detect associations driven by moderate-to-advanced CKD. Thus, our findings should not be read as evidence that eGFR has no relationship with DR in all diabetic populations. Rather, they support the more specific conclusion that, within the kidney function range represented in these cohorts and after joint adjustment for albuminuria, eGFR did not provide independent explanatory information for DR severity or OCTA-derived ischemic burden. Larger cohorts enriched for reduced eGFR are needed to determine whether filtration loss adds retinal risk information in later-stage CKD.

The study design also adds an important robustness test. The discovery and replication cohorts differed materially in the case mix, baseline risk, grading framework, and data structure; however, the direction and relative specificity of the UACR signal were conserved. That concordance argues against the association being an artifact of a single dataset or grading schema. The imaging bridge further sharpens the interpretation by showing that the systemic renal signal maps not only to ordinal DR severity but also to directly quantified capillary non-perfusion, a phenotype more proximally linked to retinal tissue jeopardy.

An additional consideration is the platform dependence of the OCTA-derived non-perfusion metrics. All OCTA scans in the present study were acquired on a single TowardPi 400k platform and processed using TP-OCTA software version 2.8.1. Although this single-platform design reduced the within-study acquisition and processing heterogeneity, the absolute non-perfusion area and perfusion density values may vary across OCTA devices due to differences in the scan geometry, sampling density, motion correction, segmentation boundaries, decorrelation or flow detection thresholds, and proprietary non-perfusion algorithms. We did not perform cross-platform calibration against Optovue, Zeiss, or other commonly used OCTA systems. Therefore, the OCTA effect sizes and absolute non-perfusion area values should be interpreted primarily as TowardPi/TP-OCTA-derived results under the present acquisition protocol. Future studies should validate whether the UACR–OCTA association is preserved across devices and whether device-specific conversion, harmonized segmentation, or common non-perfusion thresholds are required.

From a translational perspective, the OCTA findings may be most relevant in the window before destructive treatment, dense media opacity, or severe structural distortion obscures the native microvasculature. The pragmatic imaging exclusions limit generalizability to late-stage treated eyes, but they also isolate a clinically meaningful group in whom retinal ischemia remains measurable and potentially modifiable. Within this window, a higher UACR identified eyes with greater non-perfusion and lower perfusion density, suggesting that albuminuria could help flag patients who may warrant closer retinal evaluation even before overt treatment-stage disease is evident (20).

Several limitations deserve emphasis. Firstly, the cross-sectional design precludes causal or temporal inference. Secondly, albuminuria was based on a single spot urine sample, introducing biologic variability that would more likely attenuate than inflate associations. Thirdly, complete covariate harmonization across cohorts was not possible, particularly for diabetes duration. Diabetes duration was directly and systematically recorded in part 2 and was therefore included in the clinical and OCTA models, whereas it was not incorporated into the primary NHANES models because a uniformly measured, design-consistent duration variable was not available across the full retinal photography cohort. This discrepancy limits direct comparison of the effect sizes between the NHANES and clinical cohorts. The smaller NHANES estimate per doubling of ACR should therefore not be interpreted as directly comparable with the larger part 2 estimate per doubling of UACR. Residual confounding by the unmeasured diabetes duration in the NHANES could have influenced the ACR estimate; however, the direction of this bias cannot be determined reliably in a cross-sectional design and could have attenuated or inflated the association. Accordingly, we interpret the two cohorts as showing directional and biological concordance rather than quantitatively interchangeable ORs. Finally, the single-center OCTA set was selectively enriched for younger, more gradable, and less treated eyes, which may limit generalizability to advanced treated disease. Nonetheless, the persistence of the association across cohorts with different structures and across multiple sensitivity analyses supports the stability of the main finding. Broader CKD prognostic and phenotype studies also underscore that albuminuria and reduced eGFR do not always travel together and may encode different risk information across diabetic populations (21–23). Several longitudinal diabetic cohorts further suggest that albuminuria is linked to future DR progression or that remission of albuminuria accompanies a lower risk of advanced retinal outcomes, supporting the need for prospective validation of UACR as a retinal biomarker (24–27). A particularly informative next step would be prospective longitudinal cohorts with repeated urine and OCTA measurements to test whether within-person UACR worsening, stabilization, or remission tracks a contemporaneous change in the macular perfusion density and widefield non-perfusion over time.

These results support a closer integration of renal and retinal risk assessment in diabetes. Contemporary ophthalmic guidance and forward-looking reviews increasingly emphasize risk-based surveillance and multimodal imaging in DR care (28, 29). Emerging OCTA literature further indicates that diabetic macular ischemia assessment, widefield OCTA detection of neovascularization, baseline non-perfusion metrics, and larger-field quantitative classification strategies may refine the staging and prognosis beyond conventional photography-based frameworks alone (30–35). If confirmed, albuminuria could serve as a practical, inexpensive biomarker for identifying patients at heightened microvascular risk across organ systems and for enriching interventional studies aimed at the prevention of diabetic retinal ischemic progression.

## Conclusions

Across a population-based discovery cohort and an independent clinical replication cohort, albuminuria consistently tracked greater DR severity and an ischemic OCTA phenotype, whereas eGFR did not show an independent association within the kidney function range represented in these cohorts. If confirmed prospectively, incorporating UACR into integrated diabetes complication assessment could provide a pragmatic trigger for earlier multimodal retinal evaluation and closer ophthalmic surveillance before overt treatment-stage ischemic damage develops.

## Data Availability

The publicly available NHANES datasets analyzed during the current study are available from the National Center for Health Statistics. The de-identified single-center clinical dataset, NHANES-derived analytic tables, and statistical code supporting the findings of this study are available from the corresponding author on reasonable request, subject to institutional ethics requirements and data-sharing regulations.
