# Supplementary material for: Albuminuria, but not eGFR, tracks diabetic retinopathy severity and retinal ischemia: population-based discovery, clinical replication, and OCTA evidence
Source: Front Endocrinol (Lausanne). 2026 Jun 19;17:1843774. doi: 10.3389/fendo.2026.1843774 (PMC13327914; doi:10.3389/fendo.2026.1843774)
Supplement: Supplementary file 1 [file DataSheet1.docx]

**Supplementary Materials**

Supplementary Methods

NHANES survey specification and supplementary analyses

NHANES supplementary analyses followed National Center for Health Statistics multicycle guidance. The 2005-2006 and 2007-2008 files were linked by SEQN after confirming comparability of variables across cycles. Because the discovery analysis combined MEC examination, laboratory, and retinal-photography variables rather than fasting-subsample analytes, a derived 4-year MEC weight (WTMEC4YR_use = 0.5 x WTMEC2YR within each cycle) was used with SDMVSTRA and SDMVPSU, and variance estimation relied on Taylor series linearization. Weighted estimates are therefore interpretable as nationally representative estimates for US adults with diabetes aged 40 years or older who attended the MEC and had gradable retinal photographs. The discovery-stage NHANES analysis was participant-based, used worse-eye AnyDR and SevereDR outcomes, and emphasized design-corrected inference rather than unweighted subgroup comparisons. Because both retinal imaging and the primary kidney measures were MEC-based variables, fasting-subsample weights were not used in the primary discovery analysis; this preserved the retinal-imaging sample but may have undercaptured earlier or previously undiagnosed diabetes identifiable only by fasting hyperglycemia. This appendix reports the participant-selection table, the baseline 5-level race/ethnicity distribution, race/ethnicity sensitivity analyses using the original RIDRETH1 coding, and exploratory dose-response analyses that complement the main-text models. Missingness remained minimal in the NHANES analytic cohort (eGFR n=6, systolic blood pressure n=6, HbA1c n=4; overlap allowed), so multiple imputation was not performed; because combined missingness affected fewer than 1% of the analytic cohort, meaningful bias from complete-case analysis was considered unlikely. Diabetes duration was intentionally not included in the NHANES discovery models because the broad diabetes definition included participants identified by HbA1c and others without a uniformly reliable self-reported age at diagnosis; requiring questionnaire-based duration would have selectively reduced the retinal-imaging sample. LDL cholesterol and triglycerides were also not added in NHANES because these analytes are available only in the fasting morning subsample with dedicated fasting weights, which would have materially reduced the discovery sample and changed the survey-weighted target estimand.

Exploratory spline and piecewise analyses were intended to visualize the shape of association rather than replace the prespecified regression framework. Accordingly, inferential emphasis remained on survey-weighted multivariable logistic regression with albuminuria modeled continuously per doubling and, in secondary analyses, by standard clinical categories; the supplementary analyses were used to show that the discovery-stage ACR signal was not materially altered by alternate race coding or descriptive reshaping of the exposure.

Single-center data architecture and source verification

The single-center source workbook comprised a de-identified linked dataset of 180 consecutively enrolled adults with type 2 diabetes from a single-center ophthalmic specialty clinic. Written consent dates were recorded for all participants, with enrollment dates spanning 26 September 2025 to 5 March 2026 and OCTA index visits spanning 1 October 2025 to 9 March 2026. The workbook was organized into linked participant-level, eye-level, scan-level, and quality-control sheets keyed by study_id. Core demographic, laboratory, blood pressure, and medication variables were stored at the participant level; DR grading, DME status, treatment history, and eye-level eligibility were stored in the eye sheet; and separate macular and widefield OCTA sheets stored scan-acquisition parameters and exported metrics. A dedicated analysis-map sheet documented derivation of each final analysis variable, and discrepancies were tracked in a QC query log until source verification. The analysis workbook excluded direct identifiers. All 180 enrolled participants had core systemic covariates and participant-level DR grading recorded; attrition to the 168-participant clinical replication set reflected only the prespecified requirement that the closest laboratory panel fall within 30 days of OCTA.

Eye-level outcome definitions and analysis-eye selection

The clinical dataset operationalized DR with the ICDR severity scale (No DR, Mild NPDR, Moderate NPDR, Severe NPDR, and PDR). Moderate-or-worse DR was defined as Moderate NPDR or worse. VTDR was defined as Severe NPDR or PDR and/or CI-DME. DME status was recorded separately as absent, non-center-involving, or center-involving. The source workbook stored 360 eye-level records (2 eyes for each enrolled participant), and each eye record contained dedicated fields for grader 1, grader 2, adjudicator, and final consensus. The grading worksheet was separated from the participant-level laboratory sheet, so graders and adjudicators did not view UACR, eGFR, HbA1c, blood pressure, other systemic covariates, or OCTA-derived metrics during DR assignment. All participant-level models used the final consensus grade for the designated analysis eye. The analysis eye was selected using the prespecified hierarchy of worse DR, then better OCTA quality, then right eye. In tied-DR cases, prioritizing the higher-quality OCTA scan could preferentially retain the less artifact-prone and potentially slightly less ischemic eye; if so, this decision would be expected to attenuate, not inflate, the UACR-OCTA association.

OCTA protocol, endpoint selection, and image quality control

Scan-level acquisition fields recorded device model, software version, protocol, repeat number, signal score, and whether manual correction was applied. To avoid conflating protocol-dependent metrics, the primary bridge analysis was restricted to a single macular protocol (6×6 mm) and a single ultrawidefield protocol (150-degree UWF), both acquired on TowardPi 400k hardware using TP-OCTA v2.8.1 export files. Final inclusion required a final quality-pass designation after application of manufacturer signal criteria and manual review of motion artifact, decentration, and segmentation integrity. Macular scans additionally required acceptable superficial, deep, and choriocapillaris segmentation and foveal centration, whereas widefield scans additionally tracked gradable area and overall segmentation. Manual-correction flags were retained in the structured dataset for QC auditing. OCTA quality review and scan-acceptance decisions were made from de-identified scan-level files without access to the participant-level laboratory sheet or the final eye-level clinical DR stage. Across the full source workbook, 302 macular scans and 304 widefield scans met final quality-pass criteria, providing the pool from which eye-level analysis sets were derived. The prespecified widefield primary endpoint was total nonperfusion area, defined as the TowardPi-exported total capillary nonperfusion area (mm²) from the widefield analysis module after quality control and any necessary limited segmentation correction; no manual hand-drawn delineation of nonperfusion was used. The prespecified main secondary macular endpoint was superficial capillary plexus parafoveal perfusion density.

Single-center statistical framework and sensitivity analyses

UACR used the directly reported laboratory mg/g value from a single random spot-urine specimen, matching the NHANES sampling framework, and was analyzed primarily as log2(UACR) and secondarily in clinical categories (<30, 30-299, and ≥300 mg/g). The continuous per-doubling models were the prespecified primary inferential basis because they preserved full-sample information and reduced instability from sparse extreme strata; category-based models were reserved for exploratory clinical illustration. Serum creatinine and the accompanying chemistry measures were obtained from the same institutional clinical laboratory and abstracted directly from the laboratory information system, thereby minimizing within-cohort assay heterogeneity. The available de-identified source files did not retain external proficiency-program metadata (eg, IDMS-traceability fields), so cross-cohort comparability was grounded in the shared spot-urine UACR design and creatinine-based eGFR framework rather than formal cross-laboratory recalibration. eGFR used the reported laboratory value when available and otherwise was recalculated from serum creatinine, age, and sex using the 2021 CKD-EPI creatinine equation. Systolic blood pressure was represented by the mean of available readings, and the primary covariate set comprised age, sex, diabetes duration, HbA1c, systolic blood pressure, body mass index, and eGFR. Because diabetes duration was systematically recorded for all 180 enrolled participants, it was included in all single-center adjusted models. Directly measured LDL cholesterol and triglycerides were complete in the source cohort and were added in lipid-adjusted sensitivity analyses (Supplementary Table S12, Panel E). Primary participant-level inference followed the analysis-set hierarchy. The 168-participant clinical replication set was modeled first, using multivariable logistic regression for moderate-or-worse DR and VTDR and ordinal logistic regression for ordered DR severity. The nested 134-participant pragmatic OCTA set was then modeled with covariate-adjusted linear regression for the prespecified widefield primary endpoint and the main secondary macular endpoint after z-score standardization of OCTA metrics; parallel raw-scale models using the original metric units were fit for interpretability and are reported narratively in the main text. Because the highest UACR stratum showed sparse counts and near-separation in ordinary categorical clinical models, category-based clinical analyses were reported using Firth-penalized logistic regression. The final single-center analysis sets were constructed as complete-case datasets with all prespecified covariates available, so no imputation was performed. The single-center cohort represented an availability-based consecutive sample from an ophthalmic specialty clinic accrued during the study period rather than a formally powered trial cohort; we therefore did not perform post hoc power calculations. Instead, precision was conveyed by 95% confidence intervals, and as a design-sensitivity descriptor the pragmatic OCTA set corresponded to a minimum detectable standardized UACR effect of approximately 0.06-0.07 SD for the 2 prespecified OCTA endpoints at 80% power and 2-sided α=0.05 under the observed covariate structure. To gauge potential selection introduced by OCTA eligibility and quality exclusions, we first descriptively compared the 134 included participants with the 34 clinically eligible participants excluded from the pragmatic OCTA set (Supplementary Table S12, Panel C). Diagnostic review showed no problematic multicollinearity (all variance inflation factors ≤2.41 in the clinical set and ≤1.96 in the pragmatic OCTA set), acceptable logistic calibration (Hosmer-Lemeshow P=0.865 for moderate-or-worse DR and P=0.953 for VTDR), and approximately normal OCTA residuals without extreme outliers (maximum externally studentized residual, 2.46). No formal multiple-comparison correction was applied to define the primary inferential rule; instead, interpretation emphasized the prespecified hierarchy of primary, main secondary, and supportive endpoints together with effect sizes and confidence intervals, with a conservative Bonferroni reference threshold reported only as a robustness check for the 6 principal UACR associations.

Prespecified OCTA sensitivity analyses then addressed 5 potential threats to inference: scan-quality thresholds, CI-DME-related segmentation distortion, short-term laboratory-to-OCTA timing, measured differential selection into the pragmatic OCTA set, and the fixed 1-eye sampling rule. The primary OCTA model was repeated in the strict widefield-quality, strict macular-quality, CI-DME-excluded, and ≤7-day lab-to-OCTA subsets (Supplementary Table S12, Panel A). To address possible selection introduced by OCTA exclusions, we also fit inverse-probability-of-selection-weighted OCTA models. Because recent PRP/anti-VEGF, vitrectomy, and major ocular exclusions created structural zero-inclusion strata, weighting was explicitly framed as a bias-sensitivity analysis within the 168-participant clinically eligible set rather than a full correction for all excluded eyes. A parsimonious logistic model estimated entry into the pragmatic OCTA set from age, sex, log2(UACR), eGFR, ordered DR rank, and CI-DME; stabilized weights were truncated at the 1st and 99th percentiles before being applied to the prespecified covariate-adjusted OCTA linear models. To assess whether the fixed 1-eye rule materially influenced inference, bilateral sensitivity analyses used eye-level generalized estimating equations with an exchangeable working correlation and robust standard errors so that both eyes could contribute while accounting for within-participant clustering. The stability of the estimates across these sensitivity analyses was interpreted as evidence against the primary OCTA findings being driven solely by the fixed-eye rule, CI-DME-related segmentation distortion, a small number of influential observations, or measured differential selection. Supplementary Table S12, Panel B reports the bilateral GEE models, and Panel D reports the selection-weighted sensitivity models. As a complementary unmeasured-confounding sensitivity analysis for the prespecified widefield primary endpoint, we also calculated an approximate E-value. Here, the E-value denotes the minimum RR-scale strength that an unmeasured confounder would need to have with both the exposure contrast and the OCTA outcome, conditional on the measured covariates, to explain away the observed association. The standardized beta coefficient was multiplied by log2(300/30)=3.322 to obtain the standardized mean-difference contrast, translated to the risk-ratio scale using RR≈exp(0.91×SMD), and then converted to point-estimate and lower-confidence-limit E-values. Because this transformation is approximate for continuous outcomes, it was interpreted only as a supportive robustness descriptor rather than a primary inferential criterion (Supplementary Table S13).

Supplementary Tables

**Supplementary Table S1.** Race/ethnicity distribution in the NHANES analytic cohort (RIDRETH1, 5 categories).

| **Race/ethnicity (RIDRETH1)** | **Survey-weighted N (%)** | **Unweighted n** |
| --- | --- | --- |
| Mexican American (1) | 1,151,652 (8.0%) | 193 |
| Other Hispanic (2) | 708,802 (4.9%) | 80 |
| Non-Hispanic White (3) | 9,407,277 (65.6%) | 392 |
| Non-Hispanic Black (4) | 2,338,854 (16.3%) | 272 |
| Other race / multi-racial (5) | 742,042 (5.2%) | 27 |

Values are survey-weighted N (%) with unweighted counts shown in the final column.

**Supplementary Table S2.** AnyDR model using the original NHANES 5-level race/ethnicity variable (RIDRETH1)

| **Predictor** | **Adjusted OR (95% CI)** | **P value** |
| --- | --- | --- |
| Intercept | 0.01 (0.00, 0.17) | 0.004 |
| ACR (per doubling; log2(ACR)) | 1.18 (1.09, 1.27) | <0.001 |
| eGFR (per 1 mL/min/1.73m²) | 0.99 (0.98, 1.00) | 0.172 |
| Age (years) | 1.02 (1.00, 1.03) | 0.083 |
| **Race/ethnicity (RIDRETH1, 5-level)** | | |
| Mexican American | Reference | — |
| Other Hispanic | 1.17 (0.54, 2.51) | 0.697 |
| Non-Hispanic White | 0.93 (0.49, 1.76) | 0.821 |
| Non-Hispanic Black | 1.65 (0.90, 3.00) | 0.120 |
| Other race / multi-racial | 0.19 (0.04, 0.79) | 0.033 |
| **Sex** | | |
| Male | Reference | — |
| Female (vs Male) | 0.73 (0.52, 1.03) | 0.086 |
| BMI (kg/m²) | 0.97 (0.94, 1.00) | 0.065 |
| SBP (mmHg) | 1.00 (1.00, 1.01) | 0.482 |
| HbA1c (%) | 1.54 (1.32, 1.78) | <0.001 |

Survey-weighted multivariable logistic regression model: AnyDR ~ log2(ACR) + eGFR + age + race5 + sex + body mass index + systolic blood pressure + HbA1c. Complete-case analytic sample: n=949; AnyDR events=293. Reference groups: race5=Mexican American and sex=male.

**Supplementary Table S3.** SevereDR model using the original NHANES 5-level race/ethnicity variable (RIDRETH1)

| **Predictor** | **Adjusted OR (95% CI)** | **P value** |
| --- | --- | --- |
| Intercept | 0.00 (0.00, 0.10) | 0.004 |
| ACR (per doubling; log2(ACR)) | 1.30 (1.18, 1.42) | <0.001 |
| eGFR (per 1 mL/min/1.73m²) | 0.99 (0.98, 1.01) | 0.343 |
| Age (years) | 1.00 (0.99, 1.02) | 0.628 |
| **Race/ethnicity (RIDRETH1, 5-level)** | | |
| Mexican American | Reference | — |
| Other Hispanic | 0.28 (0.12, 0.65) | 0.008 |
| Non-Hispanic White | 0.58 (0.25, 1.32) | 0.209 |
| Non-Hispanic Black | 1.31 (0.54, 3.16) | 0.553 |
| Other race / multi-racial | 0.49 (0.07, 3.43) | 0.478 |
| **Sex** | | |
| Male | Reference | — |
| Female (vs Male) | 0.88 (0.49, 1.59) | 0.685 |
| BMI (kg/m²) | 1.01 (0.96, 1.06) | 0.761 |
| SBP (mmHg) | 1.00 (0.98, 1.01) | 0.885 |
| HbA1c (%) | 1.44 (1.25, 1.66) | <0.001 |

Survey-weighted multivariable logistic regression model: SevereDR ~ log2(ACR) + eGFR + age + race5 + sex + body mass index + systolic blood pressure + HbA1c. Complete-case analytic sample: n=949; SevereDR events=94. Reference groups: race5=Mexican American and sex=male.

**Supplementary Table S4.** Participant selection and exclusions in NHANES 2005-2008.

| **Step** | **N remaining** | **Excluded at this step** |
| --- | --- | --- |
| NHANES 2005–2008 MEC participants (WTMEC4YR_use non-missing) | 20,497 | — |
| Age ≥40 years | 7,081 | 13,416 |
| Diabetes (broad definition) | 6,719 | 362 |
| Gradable fundus photographs / DR data available (matched DR dataset) | 964 | 5,755 |
| Complete-case for primary regression models (Table 2) | 949 | 15 |

This table provides the more granular participant-selection workflow underlying main-text Figure 1A. Complete-case exclusions were not mutually exclusive: eGFR missing n=6, systolic blood pressure missing n=6, and HbA1c missing n=4.

**Supplementary Table S5.** Exploratory piecewise association of ACR with AnyDR in NHANES.

| **Segment** | **OR per ACR doubling (95% CI)** |
| --- | --- |
| Below threshold (<55.3 mg/g) | 1.03 (0.91, 1.17) |
| Above threshold (≥55.3 mg/g) | 1.44 (1.24, 1.66) |

Exploratory analysis based on an estimated threshold around 55.3 mg/g. The restricted cubic spline model did not show strong evidence of nonlinearity (P for nonlinearity=0.098). The corresponding ACR spline visualization is provided as Supplementary Figure S3.

**Supplementary Table S6.** Analysis-set derivation for the single-center cohort.

| **Step** | **N** |
| --- | --- |
| Enrolled source cohort with completed DR grading | 180 |
| Complete UACR, serum creatinine/eGFR, HbA1c, and blood pressure available | 180 |
| Clinical replication set: closest laboratory panel within 30 days of OCTA | 168 |
| Excluded from clinical replication because closest laboratory panel was >30 days from OCTA | 12 |
| Pragmatic OCTA set: no ocular exclusion, no recent PRP/anti-VEGF, widefield endpoint available | 134 |
| Strict widefield quality-pass set | 126 |
| Strict macular quality-pass set | 123 |
| Pragmatic OCTA set excluding center-involving DME | 125 |

Counts are participant-level and based on the prespecified analysis-eye dataset. All 180 enrolled participants had UACR, serum creatinine/eGFR, HbA1c, systolic blood pressure, and participant-level DR grading recorded; the 12-participant reduction from 180 to 168 reflected only the preplanned requirement that the closest laboratory panel fall within 30 days of OCTA (range among excluded participants, 32-57 days).

**Supplementary Table S7.** Exclusion reasons from the pragmatic OCTA set.

| **Exclusion reason** | **N** |
| --- | --- |
| anti-VEGF within 3 months | 10 |
| PRP within 6 months | 8 |
| vitrectomy history | 4 |
| significant media opacity; poor OCTA quality | 3 |
| poor OCTA quality | 3 |
| anti-VEGF within 3 months; vitrectomy history | 2 |
| anti-VEGF within 3 months; poor OCTA quality | 2 |
| significant media opacity | 1 |
| significant media opacity; vitrectomy history; poor OCTA quality | 1 |

These are mutually exclusive exclusion patterns among participants who met the 30-day laboratory-window criterion but were not included in the pragmatic OCTA set.

**Supplementary Table S8.** Participant characteristics by UACR category in the pragmatic OCTA set.

| **UACR category** | **N** | **Age, years** | **Male sex** | **Diabetes duration, years** | **BMI, kg/m²** | **SBP, mmHg** | **HbA1c, %** | **eGFR, mL/min/1.73 m²** | **UACR, mg/g** |
| --- | --- | --- | --- | --- | --- | --- | --- | --- | --- |
| <30 | 60 | 53.8 ± 9.8 | 29 (48.3%) | 7.3 ± 4.2 | 26.9 ± 3.6 | 129.6 ± 9.7 | 7.8 ± 1.1 | 95.9 ± 13.7 | 15.0 (11.0-19.2) |
| 30-299 | 49 | 55.2 ± 9.5 | 26 (53.1%) | 8.1 ± 4.7 | 25.7 ± 3.8 | 130.7 ± 11.1 | 7.8 ± 1.0 | 92.4 ± 14.1 | 91.0 (71.0-138.0) |
| >=300 | 25 | 54.7 ± 9.0 | 9 (36.0%) | 8.5 ± 4.1 | 24.9 ± 3.0 | 133.0 ± 12.4 | 8.2 ± 1.0 | 82.7 ± 15.6 | 491.0 (336.0-850.0) |

Values are mean ± SD, median (IQR), or n (%), as appropriate.

**Supplementary Table S9.** Retinal outcomes and OCTA metrics by UACR category in the pragmatic OCTA set.

| **UACR category** | **N** | **Moderate-or-worse DR** | **VTDR** | **PDR** | **CI-DME** | **Widefield total nonperfusion area** | **Macular SCP parafoveal PD** |
| --- | --- | --- | --- | --- | --- | --- | --- |
| <30 | 60 | 15 (25.0%) | 3 (5.0%) | 1 (1.7%) | 1 (1.7%) | 3.4 ± 2.6 | 50.9 ± 1.6 |
| 30-299 | 49 | 27 (55.1%) | 13 (26.5%) | 2 (4.1%) | 2 (4.1%) | 7.2 ± 3.9 | 48.4 ± 2.1 |
| >=300 | 25 | 21 (84.0%) | 13 (52.0%) | 7 (28.0%) | 6 (24.0%) | 12.3 ± 3.7 | 46.0 ± 1.9 |

This table provides the descriptive category gradient underlying the prevalence and boxplot figures in the main manuscript.

Supplementary Table S10. Exploratory Firth-penalized category-based UACR models for clinical outcomes.

| Outcome | Contrast | Adjusted OR (95% CI) | P value | N |
| --- | --- | --- | --- | --- |
| Moderate-or-worse DR | UACR 30-299 vs <30 mg/g | 11.22 (3.04-41.44) | <0.001 | 168 |
| Moderate-or-worse DR | UACR ≥300 vs <30 mg/g | 60.68 (8.27-445.21) | <0.001 | 168 |
| VTDR | UACR 30-299 vs <30 mg/g | 24.39 (4.83-123.08) | <0.001 | 168 |
| VTDR | UACR ≥300 vs <30 mg/g | 61.94 (7.56-507.34) | <0.001 | 168 |

Firth-penalized logistic regression was used to reduce small-sample bias and quasi-complete separation in the highest UACR stratum. All models were adjusted for age, sex, diabetes duration, HbA1c, systolic blood pressure, body mass index, and eGFR. The highest-risk stratum still comprised 34 moderate-or-worse DR events and 26 VTDR events among 38 participants, so the corresponding penalized odds ratios are best interpreted as confirming a strong monotonic gradient rather than as stand-alone effect sizes. These exploratory category models served only as clinical illustrations; the absolute prevalence gradient (27.9% to 89.5% for moderate-or-worse DR and 10.3% to 68.4% for VTDR) is more clinically interpretable than any single extreme point estimate.

Supplementary Table S11. Exploratory category-based UACR models for OCTA endpoints.

| **Outcome** | **Contrast** | **Adjusted beta, SD units (95% CI)** | **P value** | **N** |
| --- | --- | --- | --- | --- |
| Widefield total nonperfusion area (z) | UACR 30-299 vs <30 mg/g | 0.723 (0.518 to 0.928) | <0.001 | 134 |
| Widefield total nonperfusion area (z) | UACR ≥300 vs <30 mg/g | 1.688 (1.413 to 1.962) | <0.001 | 134 |
| Macular SCP parafoveal perfusion density (z) | UACR 30-299 vs <30 mg/g | -0.860 (-1.076 to -0.644) | <0.001 | 134 |
| Macular SCP parafoveal perfusion density (z) | UACR ≥300 vs <30 mg/g | -1.645 (-1.934 to -1.355) | <0.001 | 134 |

Standardized beta values indicate SD change in the OCTA endpoint relative to the reference UACR category (<30 mg/g). These category-based OCTA models are supportive clinical illustrations; primary inference rests on the continuous per-doubling models reported in the main manuscript.

**Supplementary Table S12. Integrated sensitivity analyses and selection diagnostics in the single-center cohort.**

**Panel A. Quality-, CI-DME-, and timing-restricted OCTA sensitivity models.**

| **Sensitivity set** | **Outcome** | **Exposure** | **Adjusted beta, SD units (95% CI)** | **P value** | **N** |
| --- | --- | --- | --- | --- | --- |
| Strict widefield QC | Widefield total nonperfusion area (z) | log2(UACR), per doubling | 0.319 (0.271 to 0.366) | <0.001 | 126 |
| Strict widefield QC | Widefield total nonperfusion area (z) | eGFR, per 1 mL/min/1.73 m² | 0.002 (-0.006 to 0.010) | 0.656 | 126 |
| Strict macular QC | Macular SCP parafoveal perfusion density (z) | log2(UACR), per doubling | -0.322 (-0.377 to -0.268) | <0.001 | 123 |
| Strict macular QC | Macular SCP parafoveal perfusion density (z) | eGFR, per 1 mL/min/1.73 m² | -0.001 (-0.010 to 0.008) | 0.867 | 123 |
| Exclude CI-DME | Widefield total nonperfusion area (z) | log2(UACR), per doubling | 0.326 (0.276 to 0.375) | <0.001 | 125 |
| Exclude CI-DME | Widefield total nonperfusion area (z) | eGFR, per 1 mL/min/1.73 m² | 0.002 (-0.006 to 0.010) | 0.622 | 125 |
| Exclude CI-DME | Macular SCP parafoveal perfusion density (z) | log2(UACR), per doubling | -0.326 (-0.380 to -0.272) | <0.001 | 125 |
| Exclude CI-DME | Macular SCP parafoveal perfusion density (z) | eGFR, per 1 mL/min/1.73 m² | -0.001 (-0.010 to 0.008) | 0.836 | 125 |
| Lab-to-OCTA ≤7 days | Widefield total nonperfusion area (z) | log2(UACR), per doubling | 0.225 (0.090 to 0.361) | 0.002 | 39 |
| Lab-to-OCTA ≤7 days | Widefield total nonperfusion area (z) | eGFR, per 1 mL/min/1.73 m² | -0.004 (-0.024 to 0.016) | 0.677 | 39 |
| Lab-to-OCTA ≤7 days | Macular SCP parafoveal perfusion density (z) | log2(UACR), per doubling | -0.290 (-0.400 to -0.181) | <0.001 | 39 |
| Lab-to-OCTA ≤7 days | Macular SCP parafoveal perfusion density (z) | eGFR, per 1 mL/min/1.73 m² | 0.006 (-0.010 to 0.022) | 0.467 | 39 |

Sensitivity models used the same covariate-adjusted framework as the primary OCTA models; the final block restricts analysis to participants whose laboratory panel was obtained within 7 days of OCTA.

**Panel B. Eye-level bilateral GEE sensitivity analyses using both eyes.**

| **Analysis set** | **Outcome** | **Exposure** | **Adjusted effect (95% CI)** | **P value** | **N** |
| --- | --- | --- | --- | --- | --- |
| Bilateral GEE (clinical) | Moderate-or-worse DR | log2(UACR), per doubling | 2.24 (1.80-2.79) | <0.001 | 336 eyes / 168 participants |
| Bilateral GEE (clinical) | Moderate-or-worse DR | eGFR, per 1 mL/min/1.73 m² | 1.00 (0.97-1.03) | 0.778 | 336 eyes / 168 participants |
| Bilateral GEE (clinical) | VTDR | log2(UACR), per doubling | 2.05 (1.67-2.53) | <0.001 | 336 eyes / 168 participants |
| Bilateral GEE (clinical) | VTDR | eGFR, per 1 mL/min/1.73 m² | 1.00 (0.96-1.03) | 0.786 | 336 eyes / 168 participants |
| Bilateral GEE (OCTA) | Widefield total nonperfusion area (z) | log2(UACR), per doubling | 0.330 (0.292 to 0.368) | <0.001 | 246 eyes / 134 participants |
| Bilateral GEE (OCTA) | Widefield total nonperfusion area (z) | eGFR, per 1 mL/min/1.73 m² | 0.001 (-0.005 to 0.007) | 0.747 | 246 eyes / 134 participants |
| Bilateral GEE (OCTA) | Macular SCP parafoveal perfusion density (z) | log2(UACR), per doubling | -0.296 (-0.338 to -0.254) | <0.001 | 246 eyes / 134 participants |
| Bilateral GEE (OCTA) | Macular SCP parafoveal perfusion density (z) | eGFR, per 1 mL/min/1.73 m² | 0.001 (-0.007 to 0.009) | 0.777 | 246 eyes / 134 participants |

Clinical rows report odds ratios; OCTA rows report standardized beta coefficients. Eye-level GEE models used an exchangeable working correlation at the participant level with robust standard errors and the same covariate-adjusted framework as the corresponding primary models.

**Panel C. Comparison of clinically eligible participants included versus excluded from the pragmatic OCTA set.**

Values are mean ± SD, median (IQR), or n (%), as appropriate. Absolute standardized differences are presented as descriptive imbalance metrics without hypothesis-testing intent. Exclusions were driven by recent PRP/anti-VEGF, vitrectomy history, media opacity, or poor OCTA quality.

| **Characteristic** | **Included in pragmatic OCTA set (n=134)** | **Excluded after clinical replication eligibility (n=34)** | **Absolute standardized difference** |
| --- | --- | --- | --- |
| Age, years | 54.5 ± 9.5 | 61.9 ± 9.9 | 0.76 |
| Male sex | 64 (47.8%) | 16 (47.1%) | 0.01 |
| Diabetes duration, years | 7.8 ± 4.4 | 12.4 ± 4.2 | 1.07 |
| SBP, mmHg | 130.7 ± 10.7 | 136.1 ± 9.1 | 0.55 |
| HbA1c, % | 7.9 ± 1.0 | 8.7 ± 1.2 | 0.66 |
| eGFR, mL/min/1.73 m² | 92.1 ± 14.9 | 76.7 ± 17.9 | 0.93 |
| UACR, mg/g | 57.0 (16.3-163.5) | 146.5 (73.3-487.0) | — |
| log2(UACR) | 5.9 ± 2.1 | 7.3 ± 2.4 | 0.63 |
| UACR <30 mg/g | 60 (44.8%) | 8 (23.5%) | 0.45 |
| UACR 30-299 mg/g | 49 (36.6%) | 13 (38.2%) | 0.03 |
| UACR ≥300 mg/g | 25 (18.7%) | 13 (38.2%) | 0.43 |
| Moderate-or-worse DR | 63 (47.0%) | 29 (85.3%) | 0.81 |
| VTDR | 29 (21.6%) | 29 (85.3%) | 1.28 |
| PDR | 10 (7.5%) | 23 (67.6%) | 1.24 |
| CI-DME | 9 (6.7%) | 11 (32.4%) | 0.65 |

**Panel D. Selection-weighted sensitivity analyses for OCTA endpoints.**

Stabilized inverse-probability-of-selection weights were estimated from the 168 clinically eligible participants using age, sex, log2(UACR), eGFR, ordered DR rank, and CI-DME as predictors of entry into the pragmatic OCTA set; weights were truncated at the 1st and 99th percentiles (selection-model C statistic, 0.879; truncated weight range, 0.80-2.88; effective weighted sample size, 117.9). Weighted OCTA models used the same covariate-adjusted framework as the primary analysis. Because recent treatment and major ocular exclusions created structural non-overlap, this analysis should be interpreted as a bias-sensitivity analysis within the clinically eligible population rather than full correction for all excluded eyes.

| **Outcome and predictor** | **Primary model, β (95% CI)** | **Selection-weighted model, β (95% CI)** | **P value (weighted)** |
| --- | --- | --- | --- |
| Widefield total nonperfusion area log2(UACR), per doubling | 0.322 (0.278 to 0.366) | 0.327 (0.282 to 0.372) | <0.001 |
| Widefield total nonperfusion area eGFR, per 1 mL/min/1.73 m² | 0.002 (-0.006 to 0.009) | 0.001 (-0.006 to 0.008) | 0.756 |
| Macular SCP parafoveal perfusion density log2(UACR), per doubling | -0.318 (-0.366 to -0.270) | -0.320 (-0.367 to -0.273) | <0.001 |
| Macular SCP parafoveal perfusion density eGFR, per 1 mL/min/1.73 m² | 0.000 (-0.008 to 0.009) | 0.001 (-0.008 to 0.010) | 0.890 |

**Panel E. Lipid-adjusted sensitivity analyses in the single-center cohort.**

| **Analysis set** | **Outcome** | **Exposure** | **Lipid-adjusted effect (95% CI)** | **P value** | **N** |
| --- | --- | --- | --- | --- | --- |
| Clinical replication | Moderate-or-worse DR | log2(UACR), per doubling | 2.91 (1.80-4.72) | <0.001 | 168 |
| Clinical replication | Moderate-or-worse DR | eGFR, per 1 mL/min/1.73 m² | 0.98 (0.92-1.04) | 0.434 | 168 |
| Clinical replication | VTDR | log2(UACR), per doubling | 2.71 (1.70-4.31) | <0.001 | 168 |
| Clinical replication | VTDR | eGFR, per 1 mL/min/1.73 m² | 0.97 (0.91-1.02) | 0.244 | 168 |
| Clinical replication | Ordinal DR severity | log2(UACR), per doubling | 2.28 (1.84-2.84) | <0.001 | 168 |
| Clinical replication | Ordinal DR severity | eGFR, per 1 mL/min/1.73 m² | 0.99 (0.96-1.02) | 0.334 | 168 |
| Pragmatic OCTA | Widefield total nonperfusion area (z) | log2(UACR), per doubling | 0.321 (0.276 to 0.366) | <0.001 | 134 |
| Pragmatic OCTA | Widefield total nonperfusion area (z) | eGFR, per 1 mL/min/1.73 m² | 0.002 (-0.006 to 0.010) | 0.623 | 134 |
| Pragmatic OCTA | Macular SCP parafoveal perfusion density (z) | log2(UACR), per doubling | -0.314 (-0.364 to -0.265) | <0.001 | 134 |
| Pragmatic OCTA | Macular SCP parafoveal perfusion density (z) | eGFR, per 1 mL/min/1.73 m² | -0.000 (-0.009 to 0.008) | 0.976 | 134 |

All models were additionally adjusted for directly measured LDL cholesterol and triglycerides alongside the prespecified covariate set. Clinical rows report odds ratios for clinical endpoints and standardized beta coefficients for OCTA endpoints.

**Supplementary Table S13. Approximate E-value analysis for the primary OCTA endpoint.**

The E-value was calculated for a clinically interpretable 30-to-300 mg/g UACR contrast (10-fold increase; 3.322 doublings) using the prespecified widefield total nonperfusion coefficient. The standardized contrast was translated to the risk-ratio scale before E-value computation; because this is approximate for a continuous outcome, the results are presented as supportive robustness descriptors.

| **Primary OCTA endpoint** | **UACR contrast** | **Approx RR-scale effect (95% CI)** | **Approx E-value (point / lower 95% CI)** |
| --- | --- | --- | --- |
| Widefield total nonperfusion area | 30 vs 300 mg/g | 2.64 (2.31-3.01) | 4.72 / 4.05 |

Supplementary Figures


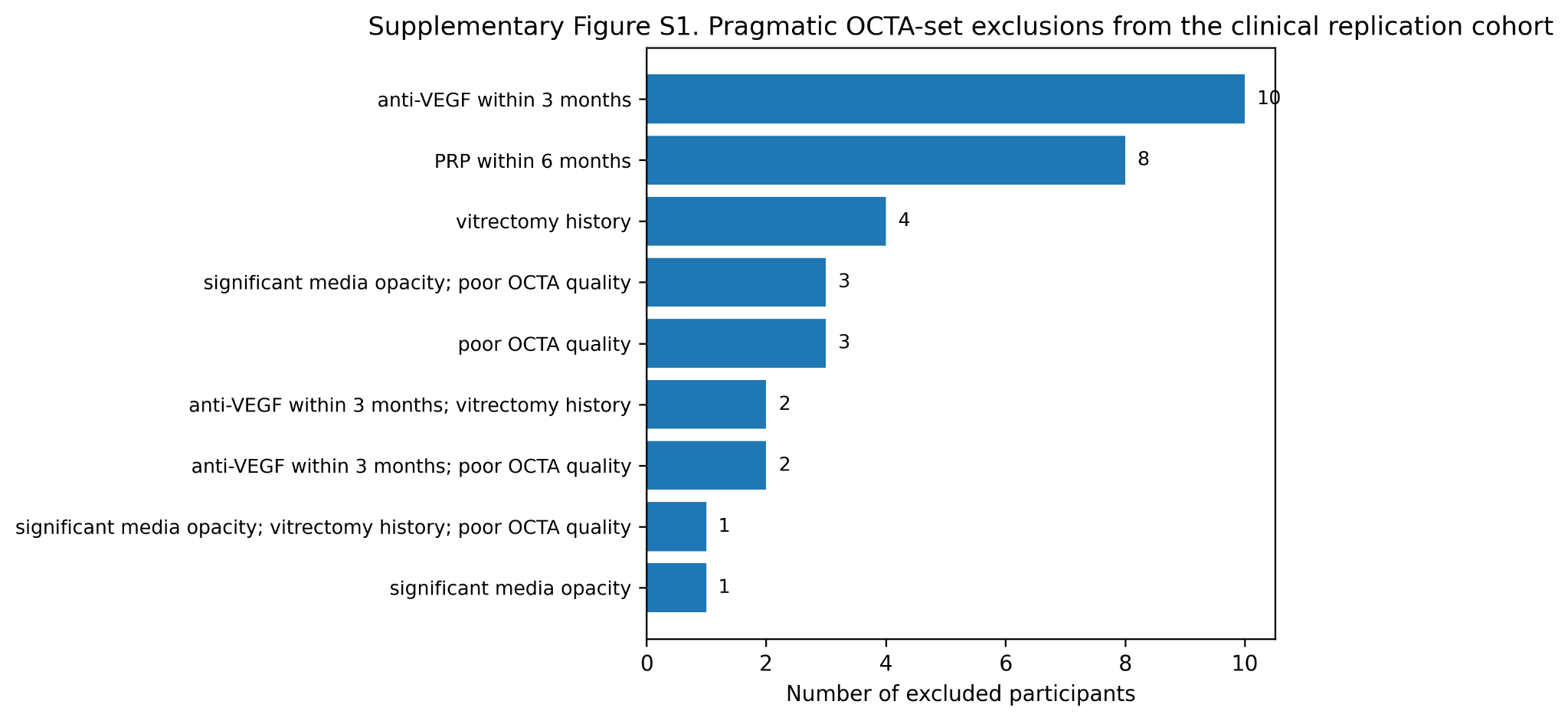


Supplementary Figure S1. Pragmatic OCTA-set exclusions from the clinical replication cohort.

The bar chart shows the frequency of each exclusion pattern among participants who met the 30-day laboratory-window criterion but did not enter the pragmatic OCTA set.


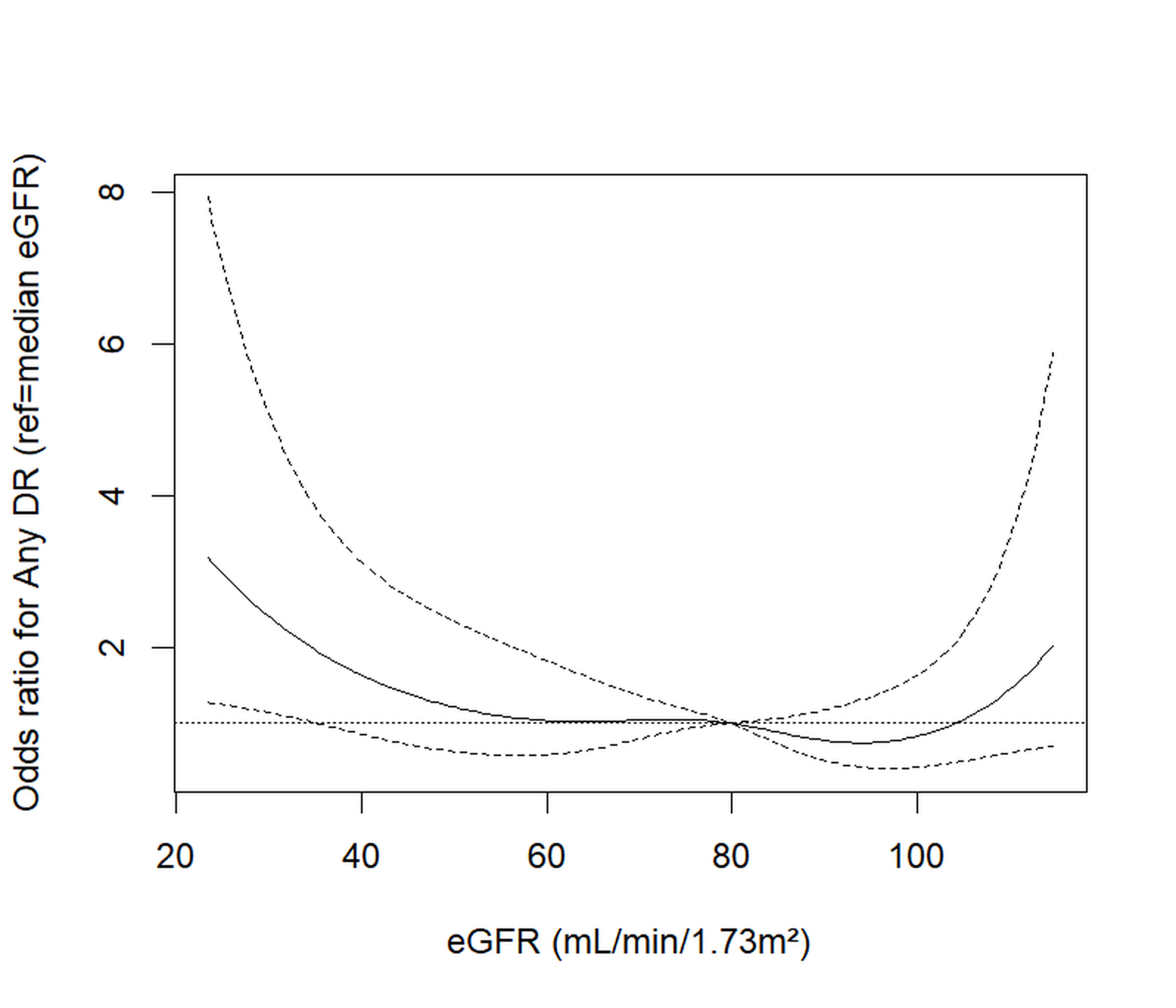


Supplementary Figure S2. Exploratory spline visualization of eGFR and AnyDR in NHANES.

The curve is displayed relative to the cohort median eGFR reference value used for centering in the spline model; for scale, the survey-weighted mean eGFR in the analytic cohort was 78.7 ± 0.9 mL/min/1.73 m². This figure is included as a descriptive visualization; the primary inferential analyses for eGFR are reported in the regression tables.


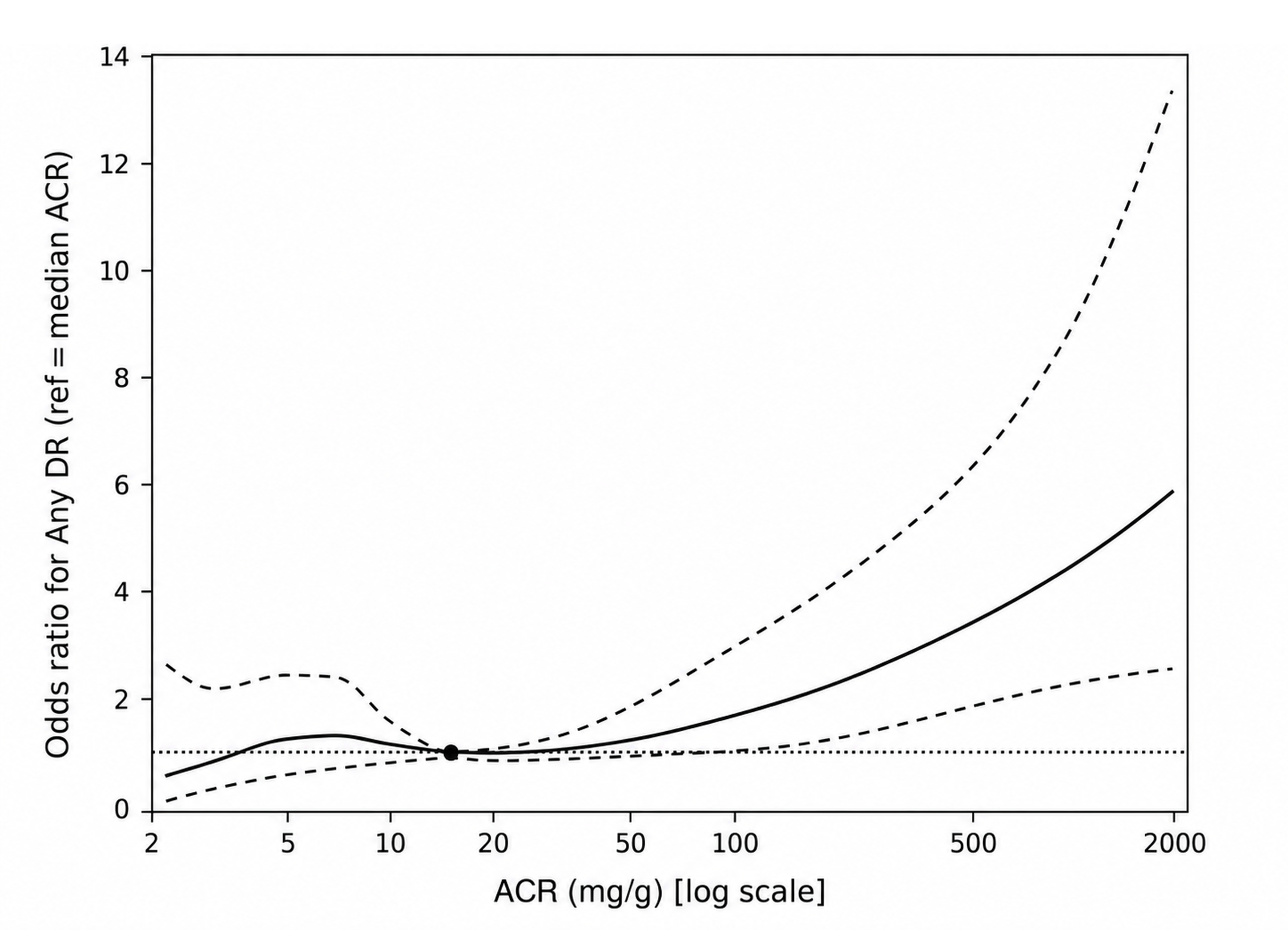


**Supplementary Figure S3. Restricted cubic spline for ACR and AnyDR in NHANES.**

Knots were placed at the 5th, 35th, 65th, and 95th percentiles. The spline did not show strong evidence of nonlinearity (P for nonlinearity = 0.098). This figure is provided as a supportive visualization after the original main-text spline figure was moved to the Supplementary Materials.
